# Integrated design process of Chinese character furniture through regular script

**DOI:** 10.1371/journal.pone.0360938

**Published:** 2026-10-26

**Authors:** Jianguo Li, Jie Shi, Yongheng Wei, Kaiqiang Yan

**Affiliations:** School of Fine Arts and Design, Hechi University, Hechi, Guangxi, China; University of Baghdad, IRAQ

## Abstract

This study proposes a design semiotics-driven, integrated innovation method for designing furniture based on Chinese regular script (Kaishu), aiming to overcome the fragmented and practice-detached approaches prevalent in current research. First, furniture-related words were collected, high-frequency terms were identified, and extensible semantic analysis was performed to select Chinese characters based on semantic flexibility. Second, the KJ method was applied to gather and analyze user needs, clarify their vector relationships, and construct a direct reachability matrix to derive a reachable matrix, resulting in structured user need analysis. User needs were then translated into functional and design requirements using Axiomatic Design (AD) theory. Third, design requirements (prioritized) were guided by shape grammar to reconstruct regular script symbols and finalize the reconstruction plan. Finally, iterative integration of product design elements achieved the commercialization of the reconstruction plan. The feasibility of this process was validated through the design example of the regular script “勤奮 (Diligence)” bookshelf, offering a novel method for expanding Chinese character furniture shapes.

## Introduction

Calligraphy, nurtured by China's millennia of cultural heritage, has evolved into diverse styles [1], becoming a quintessential symbol of Chinese cultural identity [2]. Beyond its practical origins, calligraphy transcends utility to embody aesthetic value, serving as a medium for contemplation and reflecting the distinctive principles of Eastern aesthetics [3]. This aesthetic resonance has permeated traditional Chinese craftsmanship, influencing decorative motifs and formal design [4]—a phenomenon aligning with aesthetician Zong Baihua's observation of shared aesthetic principles across Chinese art forms [5]. Notably, the linear aesthetics of Chinese calligraphy have directly shaped the silhouettes of traditional furniture, particularly during the Ming and Qing dynasties when this influence reached its zenith [6]. Ming and Qing furniture often subtly embodies calligraphic elegance [7], as seen in the “Shou” (Longevity) character chair, where the backrest's carved void abstractly mirrors the strokes of the character [8]. This integration exemplifies how calligraphic artistry transcends medium, embedding itself in functional design while preserving cultural continuity.

Driven by China's cultural heritage, modern furniture design increasingly emphasizes emotional resonance and individual expression. Against this backdrop, Chinese character furniture—a design form deeply rooted in Chinese identity—has emerged. From a categorical perspective, it aligns with the principles of new Chinese-style furniture [9], which integrates traditional cultural essence with contemporary design. New Chinese-style furniture is defined by extracting core elements from Chinese tradition [10], refining cultural symbols, and applying modern craftsmanship to create pieces that balance cultural depth with contemporary spirit [11]. Specifically, Chinese character furniture leverages calligraphic artistry, transforming two-dimensional script into three-dimensional structures through innovative design. Regular script furniture is the most prevalent type due to its clarity, structural adaptability, and broad cultural recognition.

Firstly, among the script styles of seal, clerical, regular, running, and cursive, regular script stands out as the most recently developed and widely used. Its simplified strokes and neutral, balanced aesthetic [12], make it highly accessible for information recording, cultural transmission, and innovation.

Secondly, the horizontal and vertical strokes of regular script naturally align with wooden furniture materials. Using the “Eight Laws of Yong [13]” as a framework—dot (丶), horizontal (一), vertical (丨), left slant (丿), right slant (㇏), lift (㇀), turn (𠃋), and hook (亅)—these elements can be effectively translated into furniture design:

Thirdly, the calligraphic principle of “calculating the white as black,” proposed by Qing Dynasty artist Deng Shiru, treats negative space (white) with the same care as solid lines (black). This principle provides a theoretical foundation for integrating regular script symbols into furniture design. Guided by the “Six Principles of Chinese Character Creation,” strokes are combined organically, with enclosed blank areas forming functional spaces within the furniture structure. This approach balances aesthetic and practical needs.

Owing to these advantages, the design potential of regular script has increasingly drawn attention in furniture research, inspiring the creation of innovative regular script furniture [14]. Regular script furniture leverages the stroke structure of regular script to construct its framework, establishing an isomorphic relationship between the two. This approach achieves a seamless integration of function and form, representing both a continuation and evolution of the linear modeling traditions in Chinese furniture design.

Currently, due to the geographical limitations of Chinese character usage, research on character-based furniture is primarily concentrated in domestic literature. First, a search was conducted on CNKI using the four keywords “Chinese character furniture,” “calligraphy furniture,” “regular script furniture,” and “text-imitation furniture,” yielding 53 publications. Based on the relevance of their titles and content, 34 publications were initially selected. Next, older literature was excluded, resulting in 26 publications from 2015 to the present. Finally, by merging homogeneous studies and removing those with insufficient innovation, the most representative and insightful literature was selected as follows:

Ji (2015) interpreted the design expression of Chinese character furniture from a semiotic perspective and proposed selecting characters based on cultural symbolism, such as those representing wealth and auspiciousness. This essentially constitutes a preliminary semantic screening [15]. Wu (2016) discussed the advantages of Chinese character furniture and proposed design principles that emphasize functionality, recognizability, and artistic value. It was also suggested to select characters with positive connotations, preserve the stylistic essence of character strokes in design, use characters with similar stroke structures for serialized derivations, and adopt a modular combination approach—deconstructing single characters or arranging and combining two or more characters—to construct furniture forms [16]. Zhou (2020) categorized character form design into concrete form methods (involving minimal alteration to the character) and abstract form methods (focusing on capturing the essence while allowing greater modification). Additionally, a “deconstruction and reorganization” method was introduced, which transforms character strokes into new symbols while maintaining recognizability [17]. Wang (2021) examined the relationship between Chinese characters and new Chinese-style furniture from structural and aesthetic perspectives, proposing design methods such as element representation and aestheticization, deconstruction-reorganization and modular combination, and capturing the artistic essence and three-dimensional ambiance [18]. Su (2023) addressed the lack of theoretical support in current text-imitation furniture design processes by proposing an integrated innovative design method based on extensible semantic analysis and shape grammar. This method incorporates user evaluations into the design process through extensible semantic analysis and uses shape grammar to provide a logical deduction path for furniture form evolution, offering a more rational operational framework for design practice. However, as this method involves completely deconstructing and reorganizing character strokes, although innovative in form, it detaches from the original stroke structure of the characters, making it difficult to recognize the character prototypes behind the furniture designs [19].

In terms of design cases in Chinese character furniture literature, there are quite a few. Divided into simplified and traditional characters, simplified characters include “high” character modular tables and chairs, “book” bookshelf, “its” children's chair, “shell” cabinet, “song” chair, “see” wall-mounted bookshelf, “moon” ladder, “south” chair, etc. Traditional characters include “book” bookshelf, “longevity” cabinet, etc. Through the collation and analysis of the above regular script furniture, several issues are revealed.

Overall, existing literature on Chinese character furniture has established the foundational design rationale, principles, and methodologies for this field. Its core contributions lie in: verifying the feasibility and affirming the design value of integrating Chinese characters as cultural symbols into furniture design; selecting characters based on their semantic meanings from a semiotic perspective, emphasizing their symbolic and allegorical significance; summarizing fundamental design techniques such as “concrete and abstract morphological approaches,” “structural deconstruction and reorganization,” and “modular combination”; proposing design principles that integrate functionality, recognizability, and artistic value; emphasizing the capture of the aesthetic essence of characters in furniture to evoke an artistic atmosphere within the Chinese style; and constructing an integrated methodological innovation through the combination of extensible semantics and shape grammar. These exploratory outcomes provide theoretical support, research directions, and case references for subsequent studies.

Against the contemporary backdrop of the “New Chinese Style” emerging as a design trend and the nation's continuous emphasis on cultural soft power, research on Chinese character furniture has shifted toward pursuing deeper cultural experiences and meeting diversified user needs [20]. However, from a systematic perspective, existing research methodologies exhibit fragmentation and ambiguity, reflecting a relative weakness in theoretical foundation. Fragmentation is evident in approaches that often focus on isolated stages of the design process rather than forming a coherent system. Ambiguity manifests in the lack of specific operational steps, relying heavily on designers’ subjective experience to a large extent, thereby lacking practical guidance. Current research cases tend to emphasize the presentation of individual design practices, with uneven quality. Moreover, studies primarily concentrate on formal innovation and cultural expression, failing to incorporate user needs as a driving force in the design process, which may lead to a disconnect between design outcomes and actual user requirements. This reflects the methodological fragmentation and experience-based nature of existing research on Chinese character-based furniture. There is a lack of a systematic theoretical framework and concrete operational procedures for translating Chinese character symbols into finished furniture products, which has led to lagging theoretical development and made it difficult to effectively guide innovative practice. This fundamental methodological gap directly results in the following four core issues in current design practice, which are closely related to semiotics and constrain the development of the field:

Firstly, furniture design is often confined to the original form of regular script symbols, resulting in a lack of morphological variation. Due to the two-dimensional nature of regular script symbols, some designs merely stretch these symbols along a single axis to create a three-dimensional entity. For example, the “see” wall-mounted bookshelf (Fig 1) closely resembles the regular script form of “see.” While this approach maintains high recognizability [21], it also makes the furniture appear rigid and lacking in design innovation. Furniture must effectively integrate multiple elements such as functionality (practical, cognitive, and aesthetic), material technology, and production costs, which should guide the evolution of regular script symbols.

**Fig 1:**
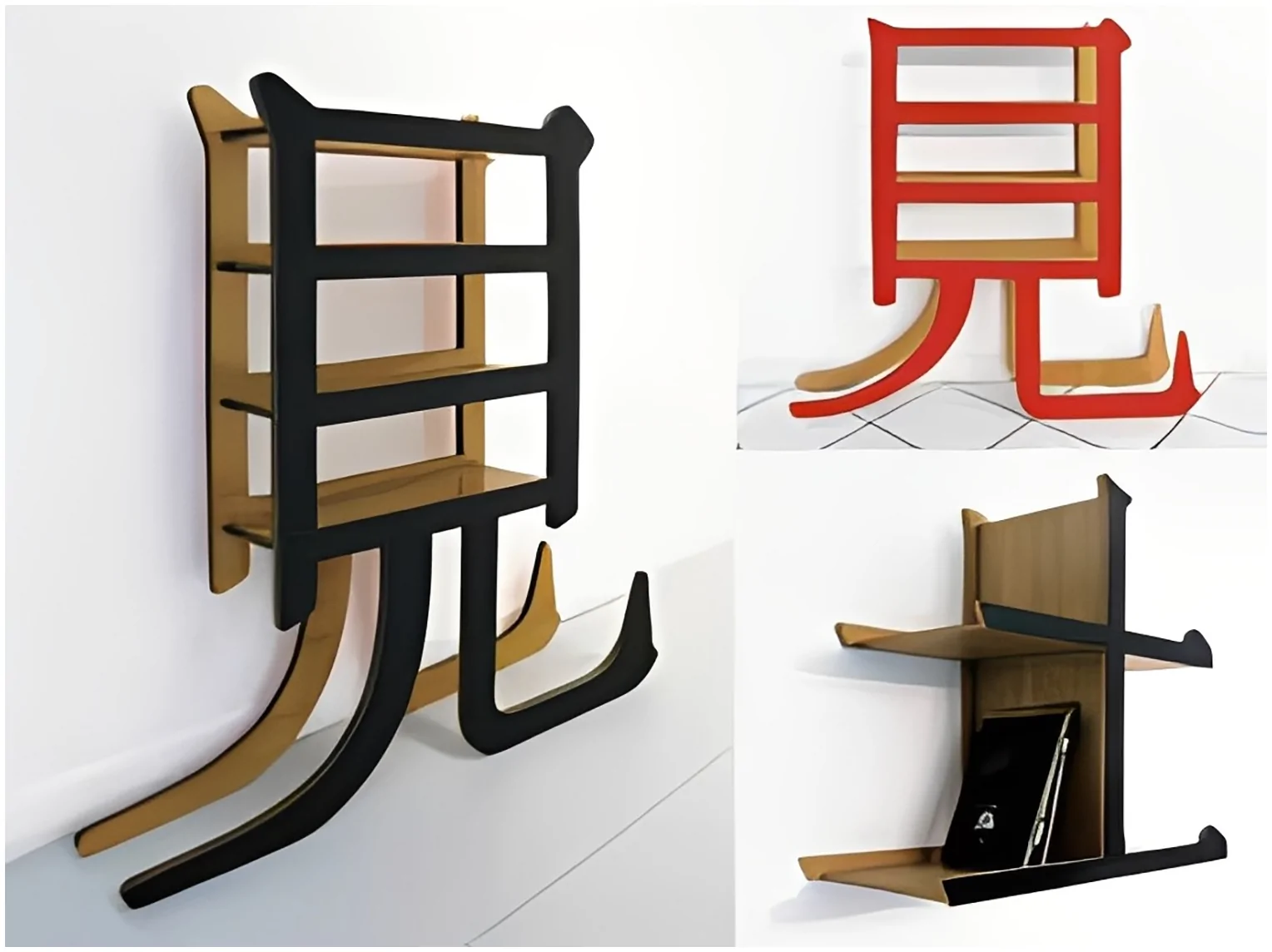
The “见(see)” wall-mounted bookshelf.

Secondly, excessive stylization in furniture shape design can obscure the recognition of its regular script prototype. Some designs fail to balance typographic form with functionality, leading to shapes that are overly altered or exaggerated, essentially losing the distinctive features of Chinese characters. This makes it difficult for viewers to discern the inherent regular script symbolic information, often resulting in the opposite of the intended effect. For instance, the “夀(longevity)” cabinet (Fig 2), with its axisymmetric block shape, abandons key character features, making it challenging to establish a metaphorical link to the “夀(longevity)” character [22].

**Fig 2:**
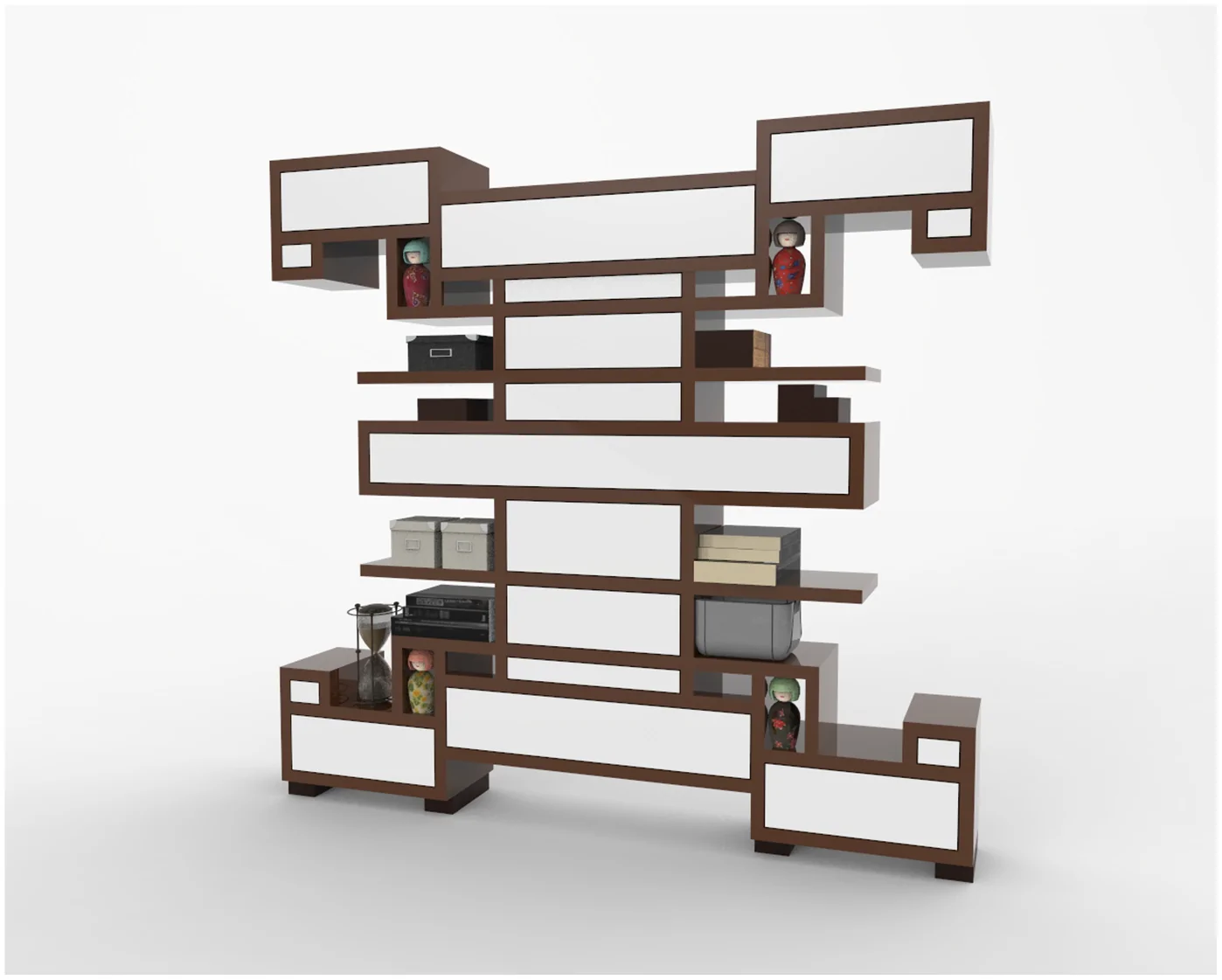
The “夀(longevity)” cabinet.

Thirdly, some furniture designs fail to convey the aesthetic nuances of regular script calligraphic techniques. Regular script calligraphy demonstrates creativity within established rules, achieving variation without rigidity, which reflects its artistic expressiveness. However, some furniture designs, due to the use of flat panel materials, reduce the shape and structure to an absolute abstraction of regular script strokes. While practical, this approach lacks the artistic charm that regular script calligraphy embodies. The “書(book)” bookshelf (Fig 3), composed entirely of flat wood panels corresponding to strokes, exemplifies this rigidity.

**Fig 3:**
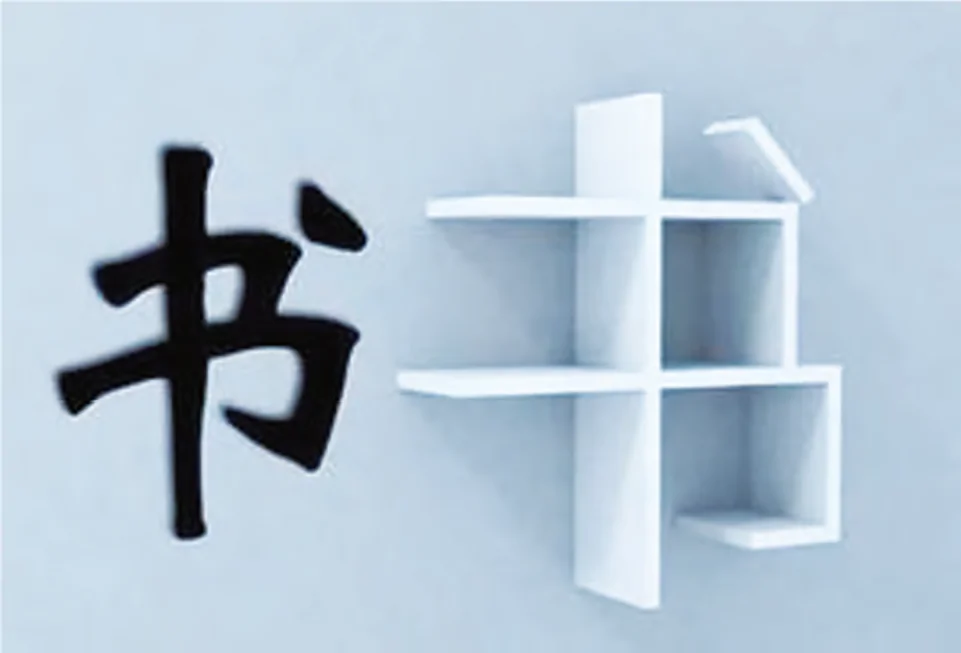
The “書(book)” bookshelf.

Fourthly, the selected Chinese characters often lack semantic association with the furniture's purpose. Research shows that character selection typically focuses on stroke structure to fulfill practical functions. However, as symbols, Chinese characters encompass both form and meaning, with the latter being crucial for establishing a semantic connection between the character and the furniture. For example, the “月(moon)” (Fig 4) ladder uses the isomorphism between the strokes of the “moon” character and the ladder's structure, but there is no meaningful association between the “moon” character and the ladder itself, making it difficult to interpret on a semantic level.

**Fig 4:**
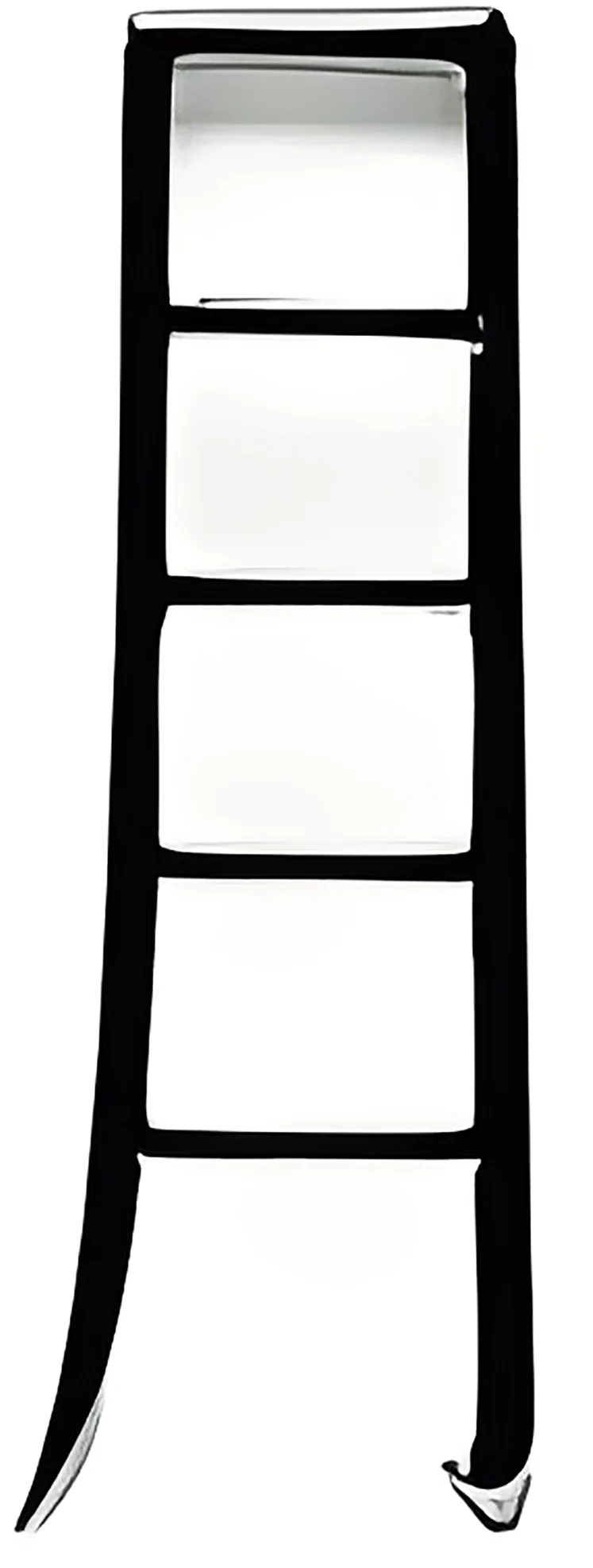
The “月(moon)” ladder.

In summary, these four issues, as manifested surface-level problems, reflect the current lack of a systematic semiotic translation methodology in the study of Chinese character furniture. Therefore, effectively addressing these issues not only corrects existing design shortcomings but also constitutes a key academic objective for advancing new Chinese-style furniture design—promoting continuous innovation in both form and meaning while ensuring its evolution with contemporary needs.

Thus, building on prior research, this paper adopts design semiotics as the analytical framework, with semantics, syntactics, and pragmatics serving as key dimensions, and selects regular script as the representative calligraphic style. It explores the process and outcome of transforming two-dimensional regular script symbols into tangible furniture forms, thereby enabling the assignment and transmission of symbolic meaning. By integrating rational research tools into existing methodological approaches, this study aims to construct a design semiotics-driven integrated design process for regular script furniture. To address the problems that existing research methods for Chinese character-based furniture are insufficiently systematic and specific and are disconnected from design practice, this study proposes a design-semiotics-driven integrated innovation design method for regular-script furniture. This method can help enhance the level of regular-script furniture design, guide the creation of furniture that both embodies distinctive ethnic cultural characteristics and meets user needs, and thereby promote the high-quality development of new Chinese-style furniture.

The proposed process initiates with regular script symbols, leveraging their stroke structures as functional frameworks, utilizing character morphology to drive aesthetic innovation, and encoding semantic depth through cultural symbolism. This multifaceted approach satisfies critical functional imperatives—practical utility, visual appeal, and educational value—while aligning with hierarchical user demands. Beyond typological expansion of Chinese character furniture, this method establishes a reference standard for new Chinese-style furniture, fostering multifunctional innovation and elevating its global market presence [23].

In the context of growing cultural pluralism, this design framework not only stimulates novel interpretations of Chinese character symbols but also provides a methodological foundation for integrating linguistic elements into everyday artifacts. By supporting the diversification and adaptive innovation of Chinese characters, this study contributes to both academic discourse and practical application in cross-cultural design.

## Materials and methods

### Related research methods

#### Design semiotics.

Design semiotics originates from semiotic theory and can be understood as the interdisciplinary application of semiotics in the field of design. Building on the work of scholars such as Ferdinand de Saussure, the American semiotician Charles W. Morris further divided semiotics into three dimensions: syntactics, which studies the structural relationships among signs; semantics, which examines the relationships between signs and their referents; and pragmatics, which investigates the structural relationships among signs, their users, and the context of use [24]. This tripartite framework has been widely adopted in the field of product design, holding significant practical value. Syntactics governs the orderly organization of formal elements; semantics influences both the immediate perception and deeper metaphors conveyed by product language [25]; while pragmatics relates to the user's experience of suitability and the visual and spatial harmony of the product within its environment.

#### Extendable semantics.

Extendable Science is an interdisciplinary field focused on resolving contradictory problems, encompassing theories and methodologies for innovation [26]. Extendable Semantics, derived from the elementary theory of Extendable Science, integrates graphical thinking and mathematical analysis to explore the relationships among physical elements, events, and relationships [27]. This method uses data analysis to quantify associations between entities, thereby uncovering the semantic information they carry. Based on the elementary theory of Extendable Science, the elementary expression is defined as follows:


$$\begin{array}{c} B=\left(O,C,P\right) \end{array}$$ (1)

where B represents the extendable element set, O represents the research object, C denotes extensible characteristics, and P signifies the quantitative value of the extendable interval.

When an object possesses multiple characteristic elements, it can be represented by a multidimensional matter-element, expressed as:


$$\begin{array}{c} \begin{array}{c} B=(O,C,P)=[\begin{array}{ccc} \begin{array}{c} O\\ \; \end{array} & \begin{array}{c} C_{\text{1}}\\ C_{\text{2}} \end{array} & \begin{array}{c} P_{\text{1}}\\ P_{\text{2}} \end{array}\\ \; & \vdots & \vdots \\ \; & C_{n} & P_{n} \end{array}] \end{array} \end{array}$$ (2)

In the equation, the research object possesses n extensible characteristics C, with each characteristic C having n corresponding extensible interval P. The magnitude P is decomposed into${\;p}_{x}$ and $p_{y}$. Here, $p_{x}$ describes the degree of cultural relevance between the research object O and the extensible characteristic C, while $p_{y}$ describes the cultural design value inherent to the extensible characteristic C.

Let the number of scores a obtained for each extensible characteristic be ${\text{𝒹}}_{a}$, where a∈[1,a] and ${\text{𝒹}}_{a}\in [\text{1},\beta ]$. Assuming the number of volunteers participating in the cultural relevance experiment is β, the formula for calculating the degree of cultural relevance between the research object O and the extensible characteristic C is:


$$\begin{array}{c} \begin{array}{c} \overset{―}{p_{x}}=\frac{\sum_{a=\text{1}}^{a}a{\text{𝒹}}_{a}}{\beta \cdot \frac{a(a+\text{1})}{\text{2}}} \end{array} \end{array}$$ (3)

Assume the valid rating given by the n-th volunteer for the cultural design value of the extensible characteristic is$\;b_{n}$, where $b_{n}\in [\text{1},\theta ]$ andn∈[1,φ], then the formula for the cultural design value of the extensible characteristic is:


$$\begin{array}{c} \begin{array}{c} \overset{―}{p_{y}}=\frac{\sum_{n=\text{1}}^{\varphi }b_{n}}{\varphi } \end{array} \end{array}$$ (4)

Based on the expressions above, the final formula for the extensible interval P is:


$$\begin{array}{c} \begin{array}{c} P=\overset{―}{p_{x}}\cdot \overset{―}{p_{y}} \end{array} \end{array}$$ (5)

In this formula, a larger P value indicates a greater extensible interval, which directly reflects the level of design value embedded in the extensible characteristic.

#### KJ method.

The KJ method, developed by Japanese anthropologist Kawakita Jiro, is a qualitative research technique used to address research problems through collaborative efforts [28]. It involves multiple research methods to gather information, which is then refined through iterative organization: ineffective information is eliminated, and similar information is merged to achieve simplification. Ultimately, solutions are derived in a graphical format. This method is particularly effective in uncovering users’ intuitive needs and is well-suited for the preliminary research phase of design. Once user requirements are identified, logical reasoning is applied to determine vector relationships among these requirements, leading to the construction of a direct reachability matrix. Through computational analysis, a reachability matrix is derived, revealing interrelationships among user requirements.

The formula for calculating the reachability matrix in the KJ method is as follows:


$$\begin{array}{c} MR=\sum_{k=1}^{N-1}A(+I) \end{array}$$ (6)

In the formula, N represents the number of effective influencing factors, and k denotes the number of variables, which is the total count of related variables in matrix A.

#### Axiomatic design.

Axiomatic Design (AD) is a structured design methodology that provides guidelines for design activities, aiming to improve design practices through systematic approaches. Axiomatic design theory establishes a mapping relationship that translates user needs into functional requirements, which are further transformed into design parameters [29]. This relationship also applies to product design drivers. For details, see Fig 5.

**Fig 5:**
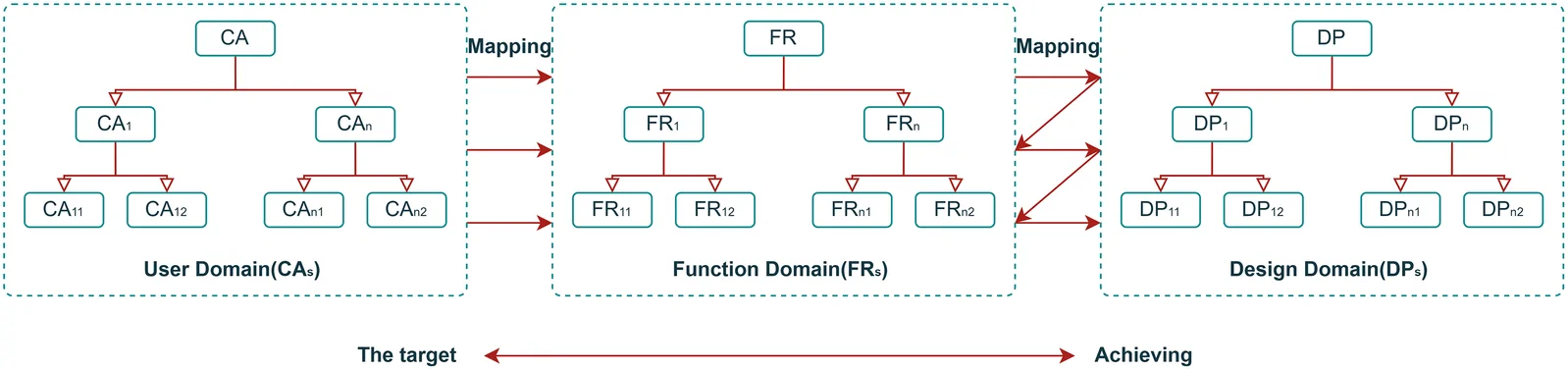
The mapping relationship of axiomatic design.

The mapping relationship between the functional domain and the design domain is represented as:


$$\begin{array}{c} \left\{FR\right\}=\left[A\right]\left\{DP\right\} \end{array}$$ (7)

Where: FR represents the vector of functional requirements, DP represents the vector of design parameters, and A represents the design matrix. Designs can be categorized into three types—non-coupled, coupled, and quasi-coupled—based on the type of matrix A [30].

#### Shape grammar.

Shape grammar, introduced by George Stiny and James Gips, operates through symbolic prototypes, using a series of syntactic rules to combine and transform symbols, thereby generating new ones. These rules, detailed in Table 1, are generative and transformational. Through iterative operations, shape grammar produces symbols with innovation, complexity, and diversity [31]. As a visual and iterative design tool, shape grammar is widely used in two-dimensional design for its efficiency and clarity. Shape grammar can be defined as:SG={S,L,R,I}, where: I represents the initial shape set; L represents a finite set of symbols; S represents a finite set of shapes; R represents a finite set of rules [32].

**Table 1:** Shape Grammar Derivation Rules.

Category	Command	Number	Illustration
**Generative Principle**	Replacement	R_1_	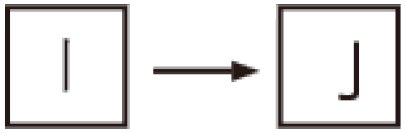
	Addition and Deletion	R_2a_ R_2b_	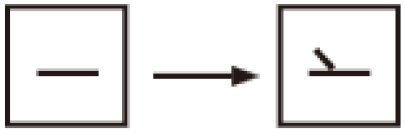
			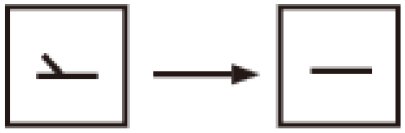
**Modifiable Principle**	Zoom	R_3_	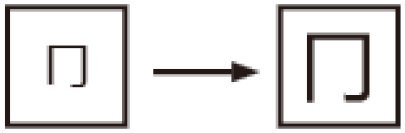
	Rotate	R_4_	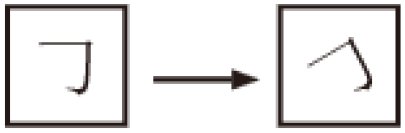
	Minor Modification	R_5_	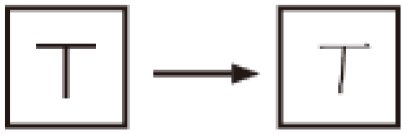
	Translation	R_6_	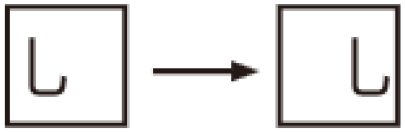
	Copy	R_7_	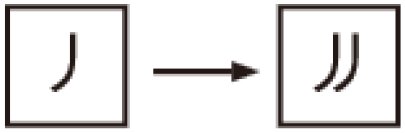
	Stretch	R_8_	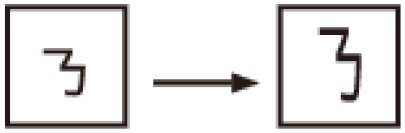

### Design process

A design process for regular script furniture has been developed in response to identified issues, based on the framework of design semiotics and integrating research methods such as extensible semantic analysis, the KJ method, axiomatic design, and shape grammar, as shown in Fig 6. This process consists of four interconnected stages from beginning to end: Chinese character screening, user requirement analysis and conversion, regular script symbol reconstruction, and design integration. The character screening stage can be understood as the semantic extension of Chinese character symbols, involving the selection of characters based on two criteria: the semantic relevance between the character and the furniture, and the character's cultural design value, aiming to establish the symbolic prototype for furniture design, which reflects the meaningful connotations and practical spatial considerations that a Chinese character should possess as a formal language for furniture. The user requirement analysis and conversion stage is driven by user needs, guiding the furniture design process to incorporate user intentions as key design factors. The regular script symbol reconstruction stage corresponds to the syntactic dimension, involving the conceptualization of the process and outcome of productizing Chinese character symbols from a two-dimensional perspective under the guidance of parameters derived from converted user requirements. The design integration stage corresponds to the pragmatic dimension, focusing on the process and outcome of productizing the reconstructed regular script symbols from a tangible, three-dimensional perspective. The above outlines the underlying logical relationships connecting these four stages, with specific details described below.

**Fig 6:**
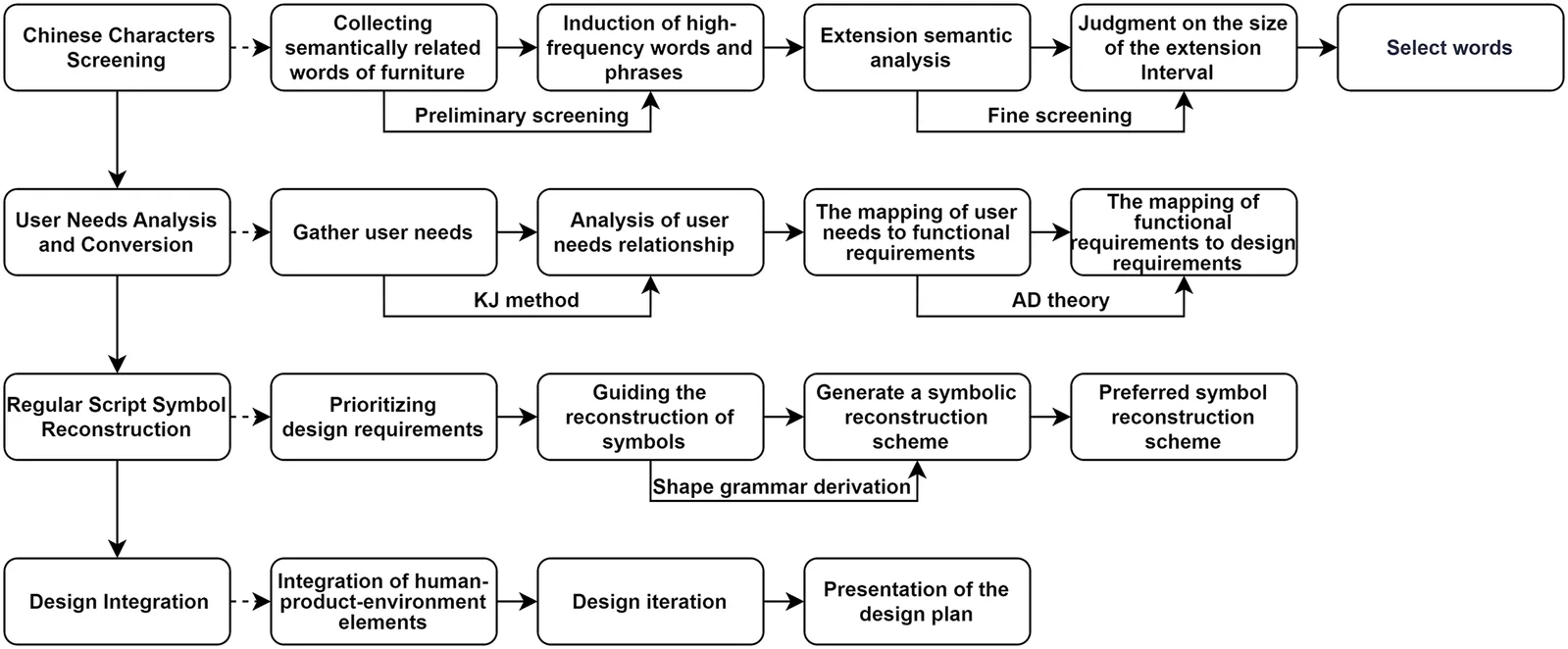
The design process of regular script furniture.

#### Chinese character screening.

Faced with the huge scale of tens of thousands of Chinese characters, the purpose of Chinese character screening is to narrow down the selection range through constraint conditions, in order to refine and finally select Chinese characters applicable to the target furniture design. Through two steps of preliminary screening and fine screening, qualitative and quantitative methods are combined to act on Chinese character screening from the semantic and syntactic levels of Chinese characters.

Preliminary screening: In response to the common method of selecting Chinese characters based on the structural characteristics of Chinese character strokes, it is proposed to collect Chinese characters from the path of semantic association, that is, through the semantic cognitive experience of the audience for the target furniture, through the association of many Chinese characters produced. These Chinese characters have a conventional semantic fit with the furniture and can reflect the humanistic value behind the furniture. These Chinese characters have a certain randomness based on commonality, depending on different educational backgrounds, life experiences, and cognitive preferences of each person. Therefore, by counting the high-frequency words that appear, the mainstream semantic cognition of the audience can be captured, which serves as the preliminary screening result of Chinese characters.

Fine screening: High-frequency words usually have several to dozens, the number varies. In order to further screen high-frequency words, the extensible semantic analysis method is used here. It is based on the extensible elementary theory, integrating semantic thinking and graphic thinking, corresponding to the implicit semantics and explicit symbols of regular script symbols, thereby converting regular script symbols into semantic elements, and then into graphic elements, in order to obtain the analysis results. According to the elementary theory in extensible learning, the calligraphy elementary can be defined as B, where B=(O,C,P), O represents regular script, C represents the high-frequency words screened, P represents the extensible interval of high-frequency words. By calculating the extensible interval values of high-frequency words, the word with the largest value is selected as the prototype for furniture design, thereby completing the fine screening of Chinese characters.

#### User needs analysis and conversion.

The KJ method, also known as the Affinity Diagram method, is a collective method for addressing research questions using various research methods to gather information and synthesize it, ultimately using a diagrammatic form to solve problems. Using the KJ method to obtain the audience's user needs for regular script furniture, the key user demand items are organized through merging and simplifying, etc. Based on logical reasoning to determine the vector relationships between user needs, a direct reachability matrix is established, and through formula calculation, a reachable matrix is derived. The analysis results point out the correlation between each demand item, obtaining the division of user's primary and expected needs, clarifying the relative importance between each demand item. Additionally, by utilizing the mapping relationship in axiomatic design, the functional requirements of furniture are derived from user requirements and subsequently translated into specific design parameters. This method enables an indirect drive of furniture design by user requirements.

#### Reconstruction of regular script symbols.

Design parameters for reconstructing regular script symbols are obtained through secondary mapping of user requirements and serve as guidelines for the reconstruction process. These parameters vary in priority, necessitating a sequential approach. Based on the two-dimensional coordinate analysis of the user requirement reachability matrix and the specific design parameter content, parameters are prioritized logically.

During the reconstruction process, strokes of regular script characters are numbered according to their writing sequence and modified using shape grammar rules, meeting designers’ needs for diverse and systematic symbol derivation. The creativity and flexibility of shape grammar tools may yield multiple reconstruction schemes compliant with design parameters. In such cases, scheme optimization is required.

#### Design integration.

Design integration involves effectively consolidating diverse design elements related to people, products, and the environment after finalizing the regular script symbol reconstruction plan [33]. This process includes transforming two-dimensional plans into basic three-dimensional entities through single-axis lofting and refining the design via multiple product iterations. As design elements integrate and overlap, the product addresses ambiguities in the reconstruction plan and is polished to near perfection with a refreshed image, reflecting the rationality of furniture design. Consequently, the furniture design plan is showcased through rendering effect drawings.

## Results and discussion

### Selecting Chinese character symbol prototypes

To gather Chinese characters semantically associated with bookshelves, a questionnaire titled “Which Chinese Characters Do You Associate with the Term ‘Bookshelf’?” was distributed to the above 73 respondents through the WeChat platform. Respondents were asked to provide three sets of characters. A total of 64 valid responses were collected, yielding 192 characters. To narrow down the selection, characters appearing only once were eliminated. The remaining characters were ranked by frequency [34]. The top five characters, with significantly higher frequencies than others, were “book” (21 times), “knowledge” (12 times), “learning” (12 times), “diligence” (10 times), and “effort” (9 times), expressed as per Formula 2.


$$\begin{array}{c} Calligraphy=\left[\begin{array}{ccc} @ccc@Regular\;script & \text{书}(Book) & p_{\text{1}}\\ & \text{知识(Knowledge)} & p_{\text{2}}\\ & \text{学习}(Learning) & p_{\text{3}}\\ & \text{勤奮}(Deligence) & p_{\text{4}}\\ & \text{努力}(Effort) & p_{\text{5}} \end{array}\right] \end{array}$$ (8)

Let the number of times each high-frequency word receives n points be denoted as $m_{n}$, where n∈[1,5] and m∈[1,16]. Then, the association value $\overset{―}{p_{x}}$ is:


$$\begin{array}{c} \overset{―}{p_{x}}=\frac{\overset{\text{5}}{\underset{n=1}{\sum }}nm_{n}}{16\cdot \frac{5\left(5+1\right)}{\text{2}}} \end{array}$$ (9)

Forty-eight calligraphy enthusiasts, aged 25–60 and experienced in regular script, participated in the survey. This age group typically encompasses working individuals who have established personal values, distinct calligraphy perceptions, and clear judgment. The participants were gathered in a relatively quiet, empty classroom, where the experimental content was presented via PowerPoint slides. First, they were briefed on the association rating, an indicator designed to quantify the strength of cultural association between candidate Chinese characters and the regular script itself. Next, the high-frequency words “书(Shu)”,“知识(Zhi Shi)”, “学习(Xue Xi)”, “勤奋(Qin Fen)” and “努力(Nu Li)” were displayed sequentially together with regular-script symbols, and participants were asked to rate the association between the high-frequency words and regular script. The association score ranged from 1 to 5 (integer values), with higher scores indicating stronger associations and lower scores indicating weaker associations. Since all these words belong to regular script, the participants unanimously agreed that they were closely associated and uniformly assigned the maximum score of 5. The calculation results based on Equation (6) are presented in Table 2.

**Table 2:** Correlation Evaluation between High-Frequency Words and Regular Script.

Regular script	“书(Shu)” Character	“知识(Zhi Shi)” Character	“学习(Xue Xi)” Character	“勤奋(Qin Fen)” Character	“努力(Nu Li)” Character
**5 points**	48	48	48	48	48
**4 points**	0	0	0	0	0
**3 points**	0	0	0	0	0
**2 points**	0	0	0	0	0
**1 point**	0	0	0	0	0
$\overset{―}{p_{x}}$	0.333	0.333	0.333	0.333	0.333

Additionally, twenty-five designers and teachers with furniture design experience and considerable knowledge of regular script were invited. These participants were gathered in a relatively quiet, empty classroom, and the experimental content was presented via PowerPoint slides. First, the cultural design value was explained to the participants, defined as the comprehensive potential of a Chinese character as a design prototype in terms of the strength of its cultural connotation, aesthetic transformation, and presupposed product function. Second, the participants were asked to evaluate the cultural design value of the above five high-frequency words. The scores ranged from 1 to 7 (integer values), with higher scores indicating greater cultural design value and lower scores indicating less. Let the score given by respondent c for a high-frequency word be ${\text{d}}_{c}$, where c∈[1,5], d∈[1,7], then the cultural design value of the high-frequency word $\overset{―}{p_{y}}$ is calculated as follows:


$$\begin{array}{c} \overset{―}{p_{y}}\;=\;\frac{\overset{\text{5}}{\underset{C=1}{\sum }}d_{C}}{\text{5}} \end{array}$$ (10)

Since some Chinese characters have both simplified and traditional forms, their cultural design values may also differ. Therefore, a comprehensive evaluation of the Chinese character forms of the high-frequency words is required. The high-frequency word forms “书(Shu)”, “書(Shu)”, “知识(Zhi Shi)”, “知識(Zhi Shi)”, “学习(Xue Xi)”, “學習(Xue Xi)”, “勤奋(Qin Fen)”, “勤奮(Qin Fen)”, and “努力(Nu Li)” were presented sequentially to the participants, who were asked to assign scores. The statistical results are presented in Table 3.

**Table 3:** Cultural Design Value Assessment of High-Frequency Words (Simplified and Traditional).

Regular script	“书(Shu)” Simplified Character	“書(Shu)” Traditional Character	“知识(Zhi Shi)” Simplified Character	“知識(Zhi Shi)” Traditional Character	“学习(Xue Xi)” Simplified Character	“學習(Xue Xi)” Traditional Character	“勤奋(Qin Fen)” Simplified Character	“勤奮(Qin Fen)” Traditional Character	“努力(Nu Li)” Character
**A1**	2	6	2	3	2	4	3	7	2
**A2**	1	6	2	2	1	3	3	7	1
**A3**	1	5	2	3	1	2	3	6	1
**A4**	3	5	2	4	3	3	3	6	2
**A5**	1	6	1	2	1	3	4	7	2
**A6**	2	7	2	3	1	4	4	6	1
**A7**	1	7	1	2	1	2	3	7	3
**A8**	2	6	1	2	2	3	3	7	2
**A9**	2	7	2	3	2	4	4	7	1
**A10**	1	6	1	2	1	3	4	7	2
**A11**	3	7	3	4	2	4	5	7	3
**A12**	2	5	3	4	2	3	4	6	1
**A13**	2	6	2	3	1	3	3	6	3
**A14**	2	7	3	4	2	4	4	7	2
**A15**	2	6	2	3	1	5	5	6	2
**A16**	1	5	2	3	2	4	3	6	1
**A17**	3	7	3	4	2	5	4	7	1
**A18**	1	4	2	2	2	3	4	5	1
**A19**	1	6	2	2	1	4	3	6	3
**A20**	2	6	2	3	1	3	4	7	1
**A21**	1	6	2	3	1	5	4	6	2
**A22**	1	5	1	1	1	3	3	6	1
**A23**	2	7	2	3	1	4	3	7	1
**A24**	3	6	3	3	2	4	5	6	2
**A25**	1	7	2	4	2	3	4	7	1
$\overset{―}{p_{y}}$	1.720	6.040	2.000	2.880	1.520	3.520	3.680	6.480	1.680

Given$P=\overset{―}{p_{x}}\cdot \overset{―}{p_{y}}$, the extensible interval for high-frequency words (simplified and traditional Chinese characters) can be calculated, as shown in Table 4.

**Table 4:** Extensible Interval Assessment of High-Frequency Words (Simplified and Traditional).

Extension interval	书	書	知识	知識	学习	學習	勤奋	勤奮	努力
$\overset{―}{p_{x}}$	0.333	0.333	0.333	0.333	0.333	0.333	0.333	0.333	0.333
$\overset{―}{p_{y}}$	1.720	6.040	2.000	2.880	1.520	3.520	3.680	6.480	1.680
p	0.573	2.011	0.666	0.959	0.506	1.172	1.225	2.158	0.559

As shown in Table 4, the extensible intervals of “book” and “diligence” rank first and second, with “diligence” having the highest value of 2.158. Therefore, the Chinese character “勤奮 (diligence)” is selected as the prototype for bookshelf design due to its greater cultural and design potential.

### Bookshelf user needs analysis and conversion

#### KJ method analysis of user needs.

User needs for regular script bookshelves were collected using the KJ method. First, given the research team’s expertise in design and cultural studies, the authors organized an internal expert workshop. Participants articulated independent viewpoints to generate demand items related to “user needs for regular script bookshelves,” resulting in a preliminary list of expert‑based need hypotheses. Second, user needs were gathered through bookshelf after‑sales feedback, online user reviews, and comments regarding bookshelves. Finally, starting from the issues identified in current regular script furniture research, the underlying problems were dissected to derive relevant user needs. Information collected from these three sources was then organized: low‑value and invalid data were removed, and similar items were merged and simplified based on affinity. For example, in hot words from buyer reviews of bookshelves on JD.com, the semantically similar terms “sturdy and durable” and “sturdy and stable” found under two different products were merged into one term based on affinity. Similarly, the hot word “rich accessories,” which pertains to evaluations unrelated to the bookshelf itself, was identified as invalid information and removed. The most representative user needs for regular script bookshelves were finally summarized, as shown in Table 5.

**Table 5:** Regular Script Bookshelf User Needs.

Number	Requested Item
**1**	Large Bookshelf Capacity
**2**	Robust and Resilient
**3**	Interestingness
**4**	Cultural Connotation
**5**	Aesthetic Sense of Modeling

The KJ method's vector relationship effectively clarifies the logical links between user requirements. User needs for bookshelves were analyzed to establish such a relationship, as shown in Fig 7. The logical reasoning is as follows: A large bookshelf capacity, which involves bearing the total weight of books, necessitates a sturdy and durable bookshelf. This capacity is directly tied to the bookshelf's size, impacting its aesthetic expression. Additionally, aesthetic appeal can stimulate the audience's interest and perception of cultural connotations.

**Fig 7:**
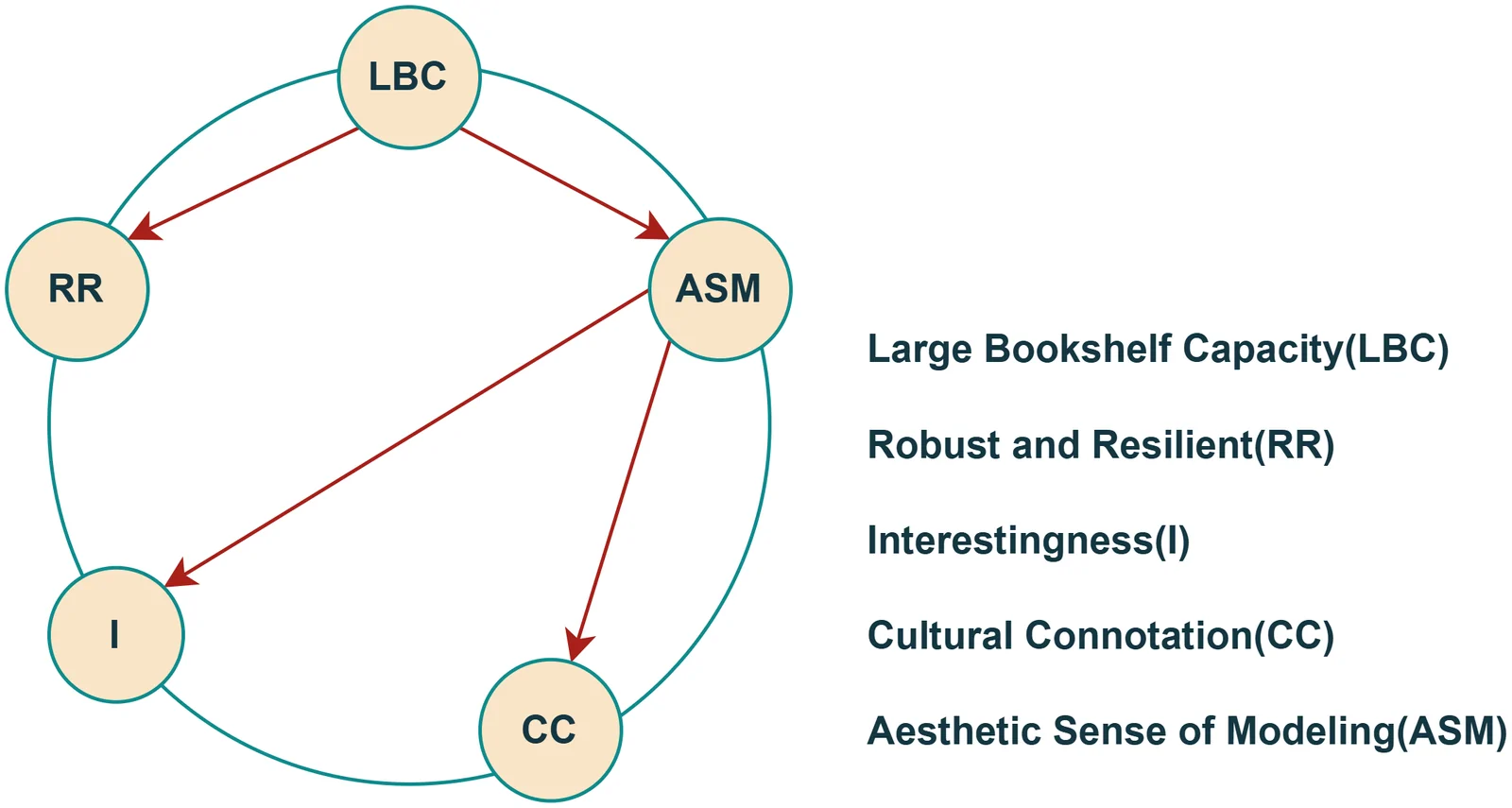
User Needs Vector Relationship.

A user needs direct reachability matrix was constructed, as shown in Fig 8. In this matrix, the influencing factors on the horizontal axis represent independent variables, while those on the vertical axis represent dependent variables. The number 1 indicates the degree of influence between the factors of the horizontal and vertical axes.

**Fig 8:**
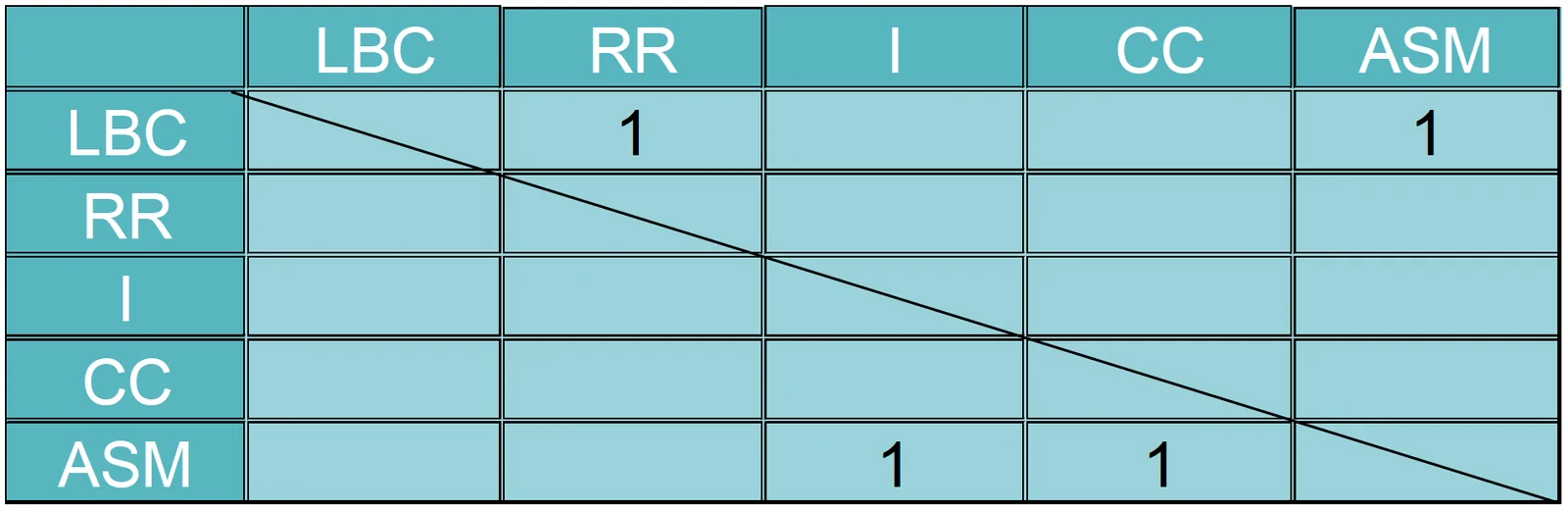
User Needs Direct Reachability Matrix.

Boolean algebra is used to calculate the direct reachability matrix by taking square roots, summing up, and deriving the reachable matrix, following Boolean algebra rules:


$$\begin{array}{c} A=\left[\begin{array}{ccccc} @lllll@0 & 1 & 0 & 0 & 1\\ 0 & 0 & 0 & 0 & 0\\ 0 & 0 & 0 & 0 & 0\\ 0 & 0 & 0 & 0 & 0\\ 0 & 0 & 1 & 1 & 0 \end{array}\right] \end{array}$$

By performing the square root, summation, and calculations on the direct reachability matrix A, the $M_{R}$ (reachability matrix) related to the problem is derived, which is given by $M_{R}=A+A^{\text{2}}+A^{\text{3}}+A^{\text{4}}+A^{\text{5}}$, with its Boolean algebra rules being:


$$\begin{array}{c} \left\{ \begin{array}{c} @c@0\times 1=0\\ 1\times 1=1\\ 0+0=0\\ 0+1=1\\ 1+1=1 \end{array}\right. \end{array}$$

Given the direct reachability matrix A, calculate $A^{\text{2}}$ using the formula:


$$\begin{array}{c} A^{\text{2}}=\left[\begin{array}{ccccc} @lllll@0 & 0 & 1 & 1 & 0\\ 0 & 0 & 0 & 0 & 0\\ 0 & 0 & 0 & 0 & 0\\ 0 & 0 & 0 & 0 & 0\\ 0 & 0 & 0 & 0 & 0 \end{array}\right] \end{array}$$

Subsequently, derive $A^{\text{3}}$, $A^{\text{4}}$, and $A^{\text{5}}$, which are all zero matrices:


$$\begin{array}{c} A^{\text{3}}=A^{\text{4}}=A^{\text{5}}=\left[\begin{array}{ccccc} @lllll@0 & 0 & 0 & 0 & 0\\ 0 & 0 & 0 & 0 & 0\\ 0 & 0 & 0 & 0 & 0\\ 0 & 0 & 0 & 0 & 0\\ 0 & 0 & 0 & 0 & 0 \end{array}\right] \end{array}$$

Finally, according to the formula calculation, the reachable matrix about the influencing factors is derived:


$$\begin{array}{c} M_{R}=\left[\begin{array}{ccccc} @lllll@0 & 1 & 1 & 1 & 1\\ 0 & 0 & 0 & 0 & 0\\ 0 & 0 & 0 & 0 & 0\\ 0 & 0 & 0 & 0 & 0\\ 0 & 0 & 1 & 1 & 0 \end{array}\right] \end{array}$$

Fill the reachable matrix in Fig 9 with the operation results, which illustrates the reachability relationships between variables of the direct matrix *A*. Here, *D* + *R* denotes the sum of independent and dependent variable factors, while *D*-*R* indicates their difference.

**Fig 9:**
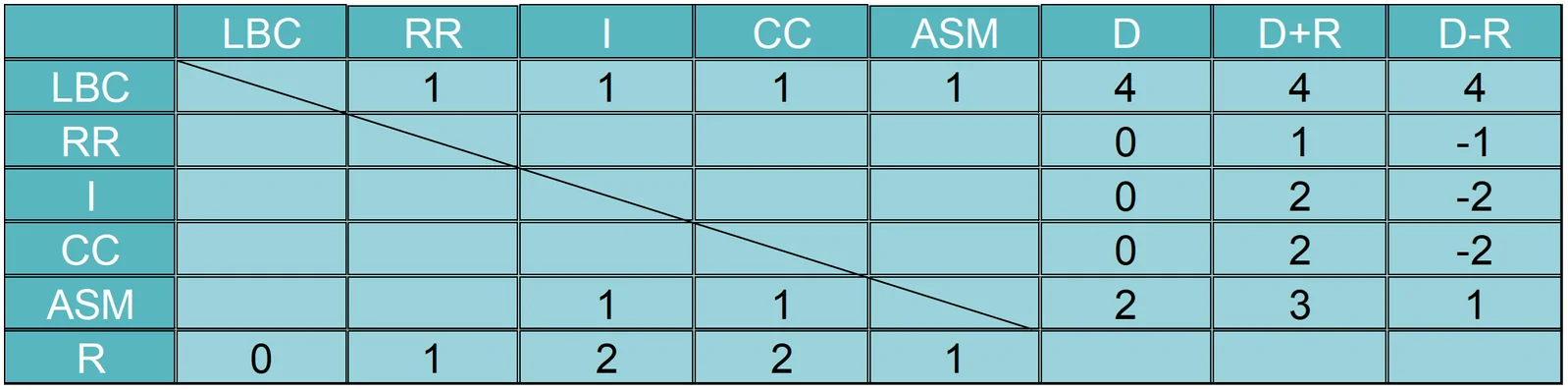
User Needs Reachable Matrix.

To visually display the correlation between user requirements, plot D + R on the horizontal axis and D − R on the vertical axis of a two – dimensional coordinate system. Map the reachable matrix results onto these coordinates. The analysis results are shown in Fig 10.

**Fig 10:**
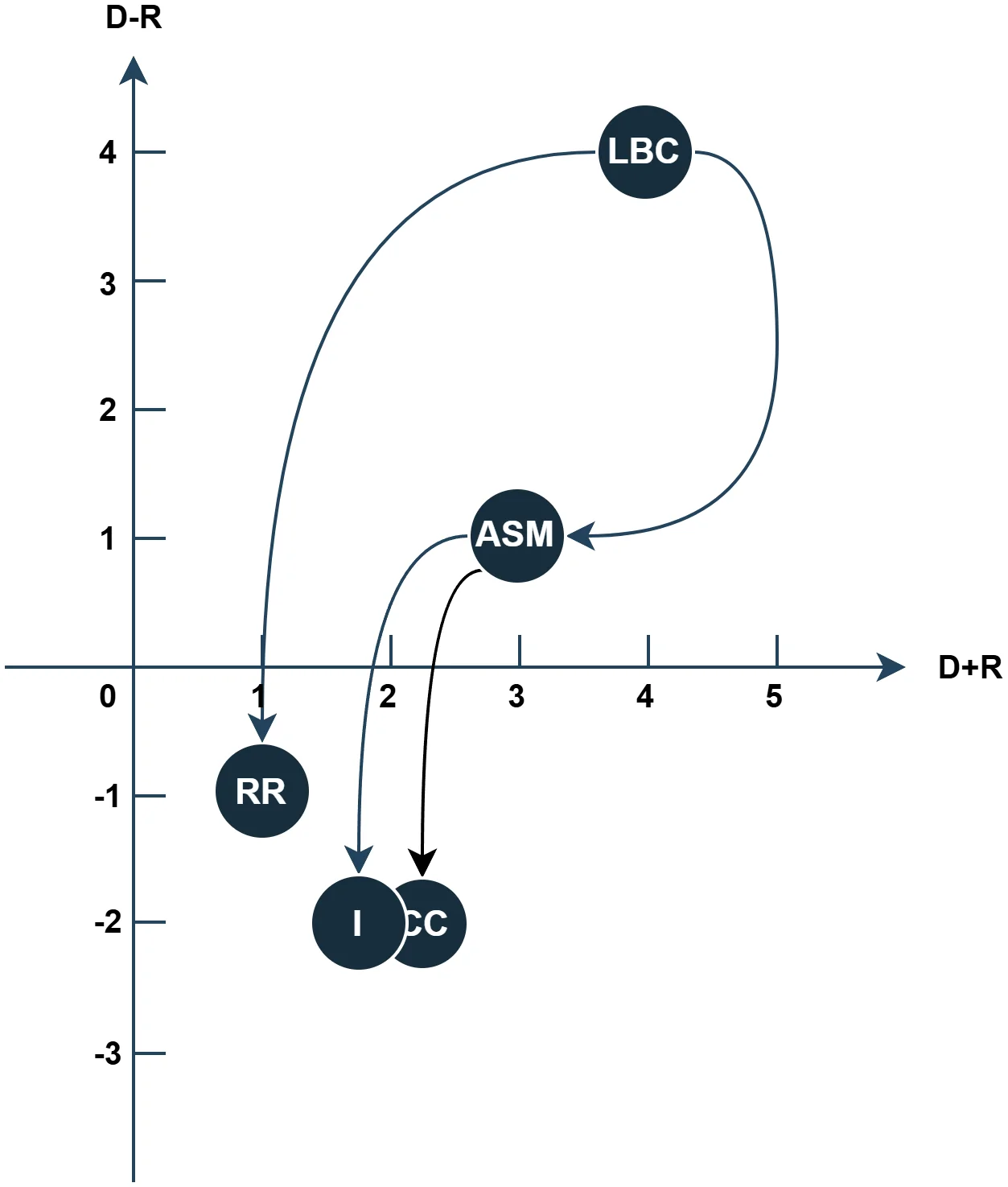
Analysis Results of User Needs.

In Fig 10, the two – dimensional coordinates of user needs show that “Book Placement Capacity” at (4,4) is the starting factor, directing to “Durability” at (1,-1) and “Aesthetic Shape” at (3,1). “Aesthetic Shape” further directs to “Fun” and “Cultural Connotation,” both at (2,-2) as end factors. This indicates “Large Bookshelf Capacity” is the primary need, while “Fun” and “Cultural Connotation” are expected needs. This reflects a hierarchical shift in user needs from practical to experiential, aligning with social development trends. These analysis results offer a reference for subsequent user – requirement conversion.

#### User needs conversion.

User requirements obtained via the KJ method are mapped to bookshelf characteristics, converting user requirements into functional requirements. Subsequently, these functional requirements are mapped to key steps in regular script symbol reconstruction. Through rational analysis, functional requirements are transformed into design requirements. This conversion of user requirements is detailed in Table 6.

**Table 6:** Conversion of User Needs.

User requirements $CA_{s}$	Functional requirements $FR_{s}$	Design requirements $DP_{s}$
Large Bookshelf Capacity $CA_{\text{1}}$	The bookshelf is of a considerable size, and the utilization rate of its functional areas is high $FR_{\text{1}}$	Strengthen the overall sense of the symbol and optimize the layout of the enclosed space of strokes $DP_{\text{1}}$
Robust and Resilient $CA_{\text{2}}$	The structure of the bookshelf is rationally stressed and the materials used are substantial $FR_{\text{2}}$	Stabilize the visual center of gravity of the symbol and optimize the stroke structure $DP_{\text{2}}$
Interestingness $CA_{\text{3}}$	The interesting experience triggered by the appearance of the bookshelf. $FR_{\text{3}}$	Detect the information redundancy of the symbol $DP_{\text{3}}$
Cultural Connotation $CA_{\text{4}}$	The cultural charm inherent in the bookshelf $FR_{\text{4}}$	Application of regular script brushwork on the symbol $DP_{\text{4}}$
Aesthetic Sense of Modeling $CA_{\text{5}}$	The aesthetic perception elicited by the appearance of the bookshelf $FR_{\text{5}}$	Strengthen the aesthetic degree of the symbol $DP_{\text{5}}$

Based on the AD theory, we obtain$\left\{FR_{\text{s}}\right\}=\{FR_{\text{1}},FR_{\text{2}},FR_{\text{3}},...,FR_{\text{n}}\}$, $\left\{DP_{s}\right\}=\{DP_{\text{1}},DP_{\text{2}},DP_{\text{3}},...,DP_{\text{n}}\}$, substituting the sets of functional requirements and design parameters into Equation (3):


$$\begin{array}{c} \left[\begin{array}{c} @c@FR_{\text{1}}\\ FR_{\text{2}}\\ \vdots \\ FR_{\text{n}} \end{array}\right]=\left[\begin{array}{cccc} @cccc@b_{11} & b_{12} & \cdots & b_{1n}\\ b_{21} & b_{22} & \cdots & b_{2n}\\ \vdots & \vdots & b_{ij} & \vdots \\ b_{n1} & b_{n2} & \cdots & b_{nn} \end{array}\right]\times \left[\begin{array}{c} @c@DP_{\text{1}}\\ DP_{\text{2}}\\ \vdots \\ DP_{\text{n}} \end{array}\right] \end{array}$$ (11)

In the formula,$b_{ij}$ the correlation degree between each element is represented. By substituting each parameter into Equation (11) for calculation, the resulting matrix is as follows:


$$\begin{array}{c} \left[\begin{array}{c} @c@FR_{\text{1}}\\ FR_{\text{2}}\\ FR_{\text{3}}\\ FR_{\text{4}}\\ FR_{\text{5}} \end{array}\right]=\left[\begin{array}{ccccc} @ccccc@X & O & O & O & O\\ O & X & O & O & O\\ O & O & X & O & O\\ O & O & O & X & O\\ O & O & O & O & X \end{array}\right]\times \left[\begin{array}{c} @c@DP_{\text{1}}\\ DP_{\text{2}}\\ DP_{\text{3}}\\ DP_{\text{4}}\\ DP_{\text{5}} \end{array}\right] \end{array}$$

In this matrix, “X ” represents a strong influence, and “O ” represents a weak influence. From the “X ” matrix, it can be seen that the elements forming a diagonal matrix indicate that the design parameters are reasonable and belong to non-coupling design [30]. This satisfies the principle of the independence axiom, and the design parameters are feasible [35].

### Reconstruction of “勤奮(qin fen)” regular script symbol

Using the Alibaba PuHuiTi L Font as an example, which has a clear structure, abstract and rigorous lines, and moderate thickness [36], it is suitable for presenting the reconstruction process of regular script symbols derived from shape grammar [37]. To facilitate observation, the Chinese character “勤奮(Qin Fen)” is horizontally dissected from left to right according to the stroke order, and each stroke is numbered, as shown in Fig 11.

**Fig 11:**
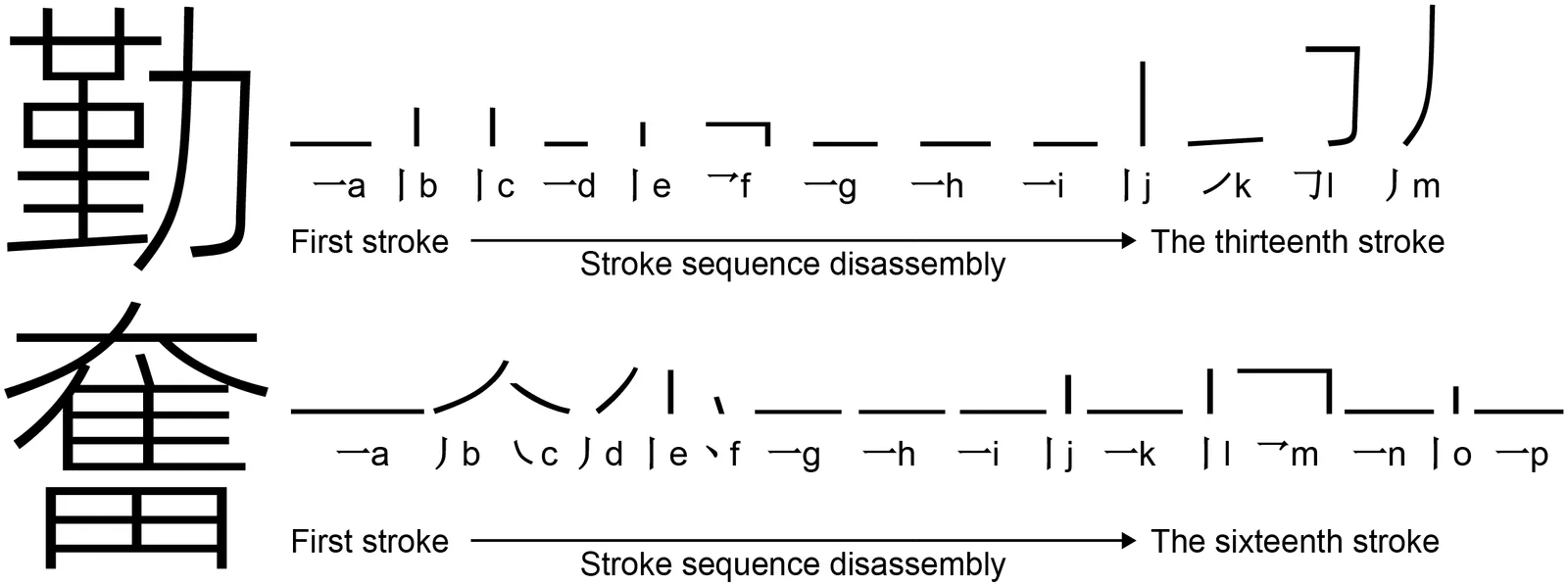
Dissection and Numbering of “勤奮(Qin Fen)” Character Strokes.

Analysis of the KJ method's user requirement plane coordinate system reveals that $DP_{\text{1}}$ is the primary design requirement, while $DP_{\text{4}}$ and $DP_{\text{3}}$ are the desired design requirements. Furthermore, based on the specific content of the design requirement items, the design requirements are prioritized in the order of $DP_{\text{1}}$ -$DP_{\text{5}}$ -$DP_{\text{2}}$ -$DP_{\text{4}}$ -$DP_{\text{3}}$, This prioritization guides the specific steps in reconstructing regular script symbols and generating multiple design schemes.

(1) To strengthen the overall sense of the symbol and optimize the layout of the enclosed stroke space ($DP_{\text{1}}$): The left side of the Chinese character is the radical, and the right side is the companion. Constructing a complete symbol typically requires forming a closed or semi-closed shape using radicals and companions. This often involves extending or shifting strokes to align or connect them, thereby expanding the enclosed stroke space. Under the guidance of $DP_{\text{1}}$, three I_2_ symbols are generated from the initial form I_1_ of the “Qin Fen” symbol, Fig 12.

**Fig 12:**
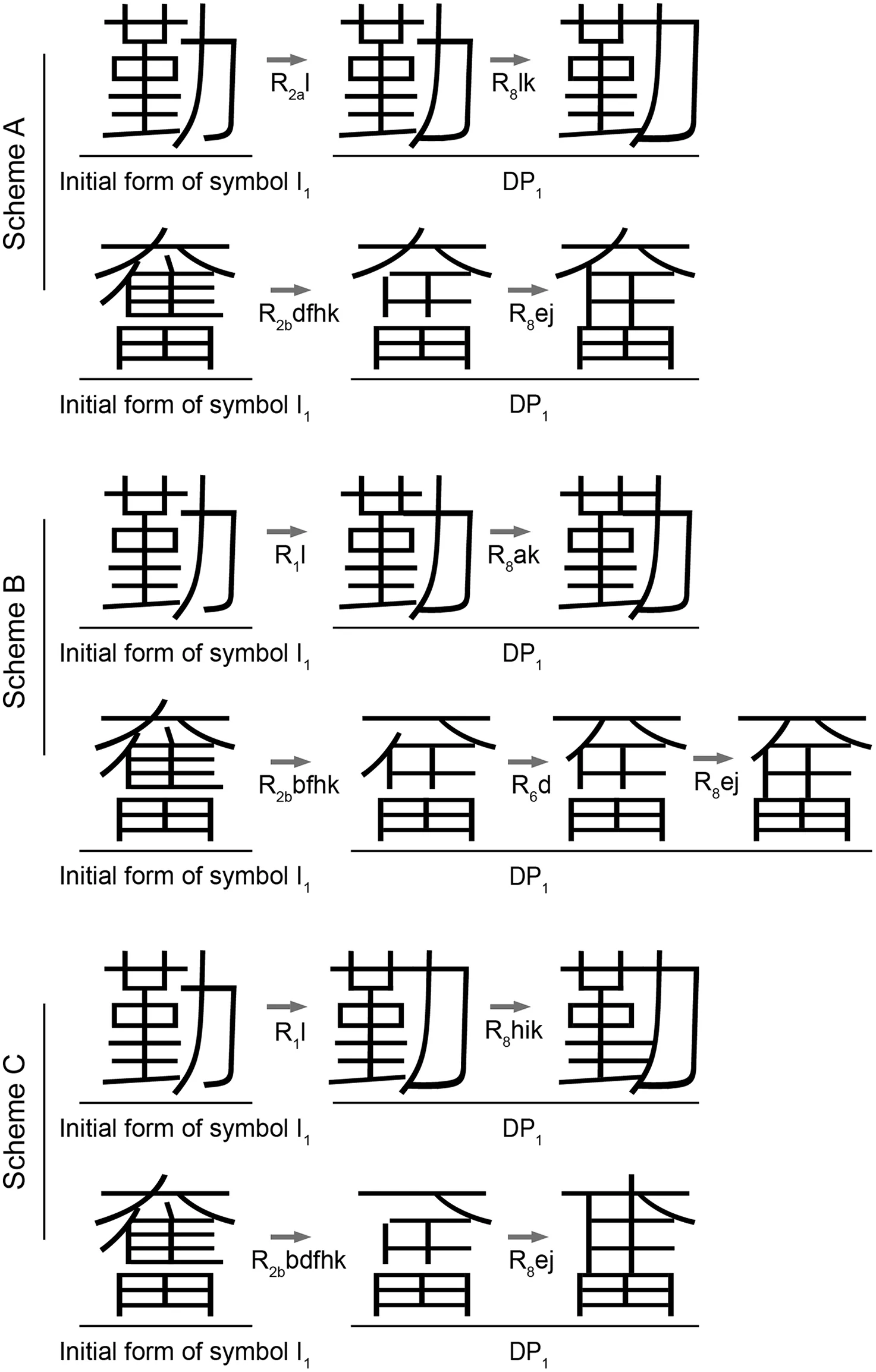
I_2_ Reconstructed Symbols Guided by $DP_{1}$.

(2) To enhance the aesthetic quality of the symbol ($DP_{\text{5}}$): it is crucial to recognize that aesthetics correlate with orderliness. Symbol harmony and regularity can be achieved by adjusting strokes of varying lengths, spacing, and directions to ensure alignment, equidistant spacing, and parallelism. Under the guidance of $DP_{\text{5}}$, three I_3_ symbols are generated from the initial form I_2_, Fig 13.

**Fig 13:**
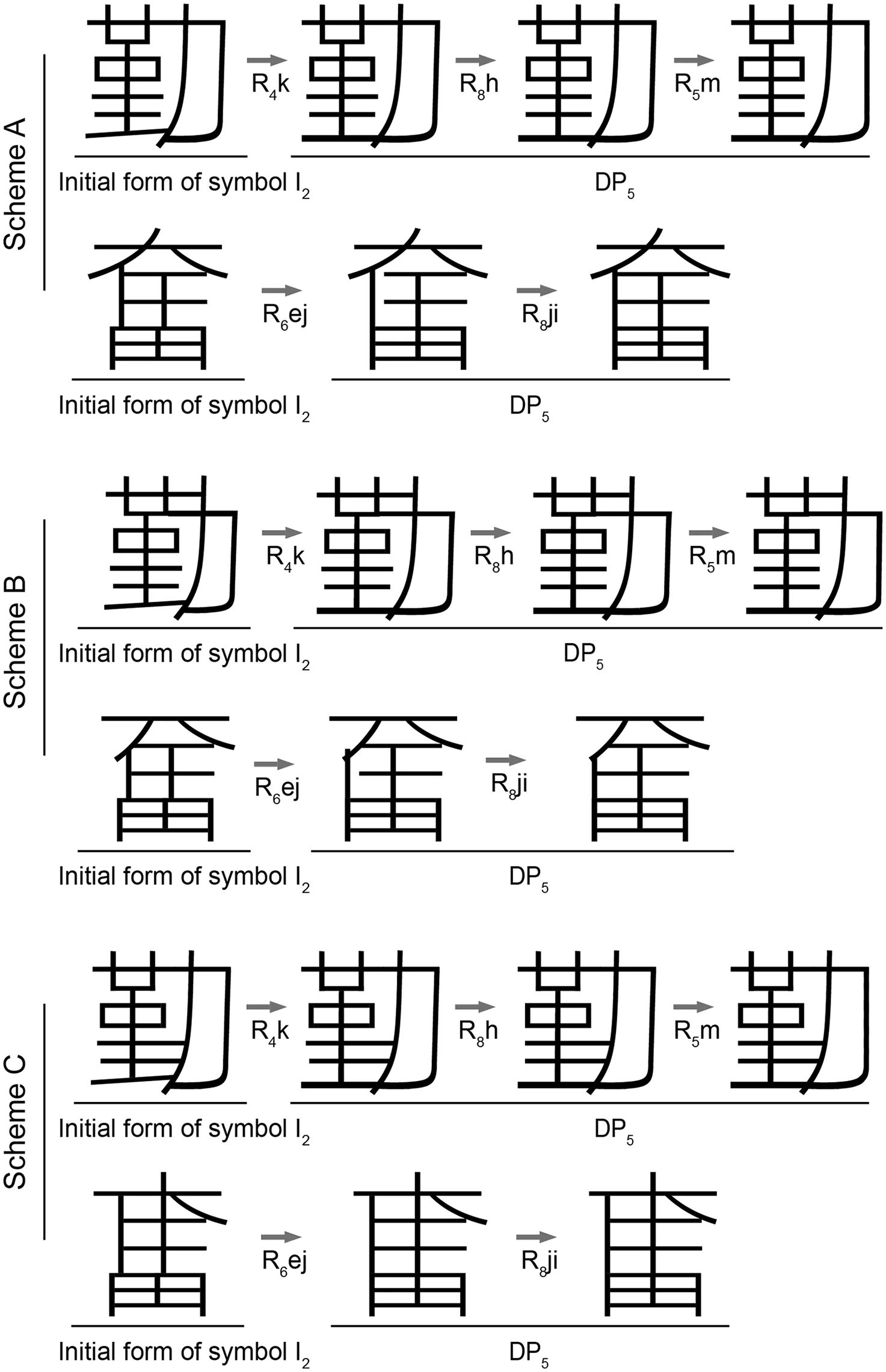
I_3_ Reconstructed Symbols Guided by $DP_{5}$.

(3) To stabilize the visual center of gravity of the symbol and optimize the stroke structure ($DP_{\text{2}}$), this design requirement corresponds to the user need for the bookshelf to be sturdy and durable. This step primarily involves modifying strokes in terms of bookshelf manufacturing materials, craftsmanship, and the rationality of the frame structure. Under the guidance of $DP_{\text{2}}$, three I_4_ symbols are generated from the initial form I_3_, Fig 14.

**Fig 14:**
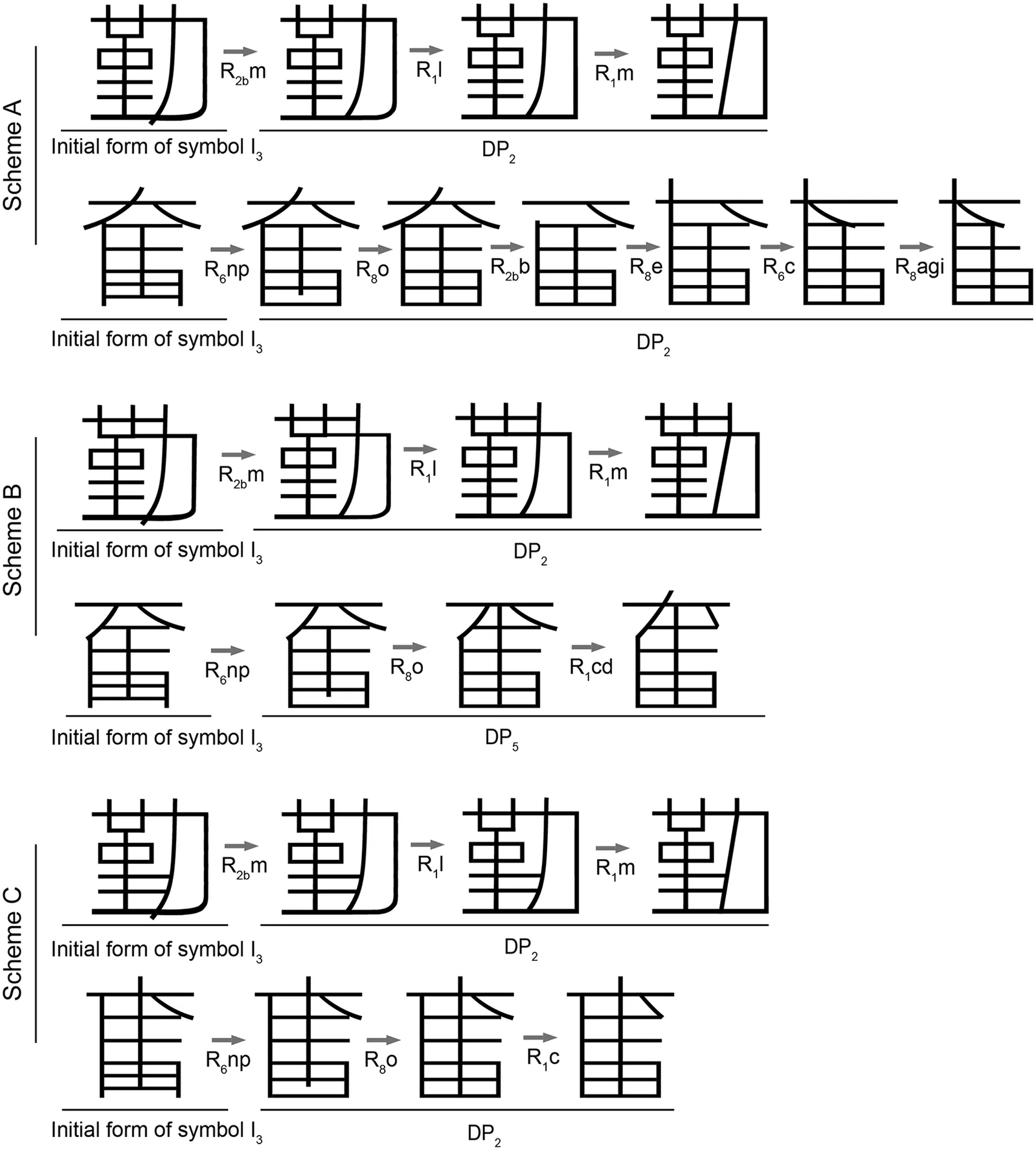
I_4_ Reconstructed Symbols Guided by $DP_{2}$.

(4) Applying regular script brushwork to symbols ($DP_{\text{4}}$): Brushwork, the essence of calligraphy, is expressed through contour variations within structural rules. Since fonts like Alibaba PuHuiTi are relatively abstract and lack traditional brushwork charm, this step selects a simple regular script font that retains key brushwork characteristics. Considering furniture manufacturing constraints, brushwork is emphasized in the symbol strokes through partial application rather than comprehensive replication. Under the guidance of $DP_{\text{4}}$, strokes from “Qin Fen” regular script are extracted and finely adjusted twice to retain essential brushwork features while increasing abstraction. These adjusted strokes replace the original strokes of the I_4_ symbol, generating three I_5_ symbol schemes, as shown in Fig 15.

**Fig 15:**
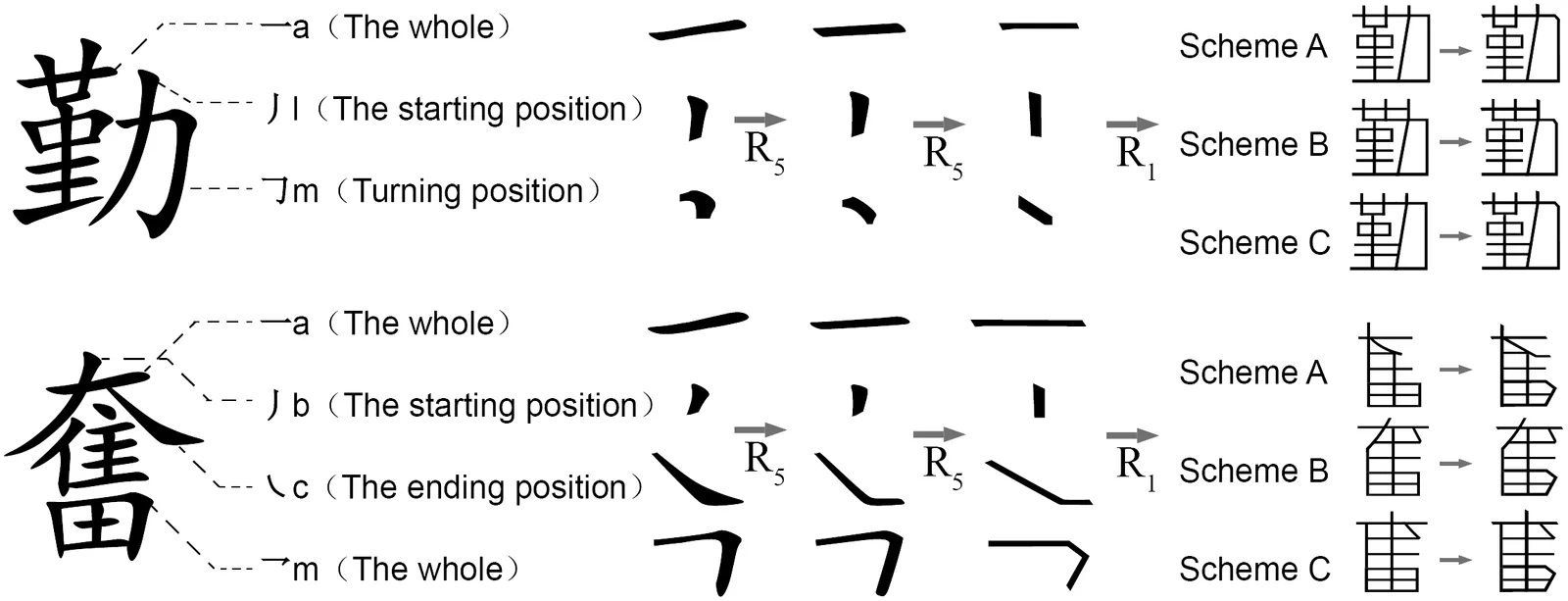
I_5_ Reconstructed Symbol Schemes Guided by $DP_{4}$.

(5) Detecting Symbol Information Redundancy ($DP_{\text{3}}$): This design requirement is linked to the user need for fun. Fun arises from the emotional experience of curiosity and recognition when the audience views symbols that “resemble regular script but are not identical.” This step checks if the reconstructed symbol contains an appropriate amount of Chinese character information redundancy to meet basic recognition requirements. Using the diagrammatic analysis of extensible semantics, the I_5_ reconstructed symbol scheme (Table 7) is evaluated for its association with the “勤奮(Qin Fen)” character to judge the Chinese character information redundancy carried by the three schemes.

**Table 7:** I_5_ Reconstructed Symbol Schemes.

“Qin Fen” Character	Scheme A	Scheme B	Scheme C
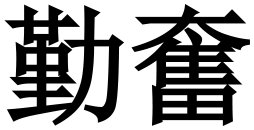	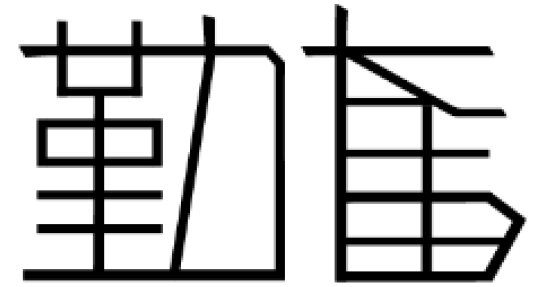	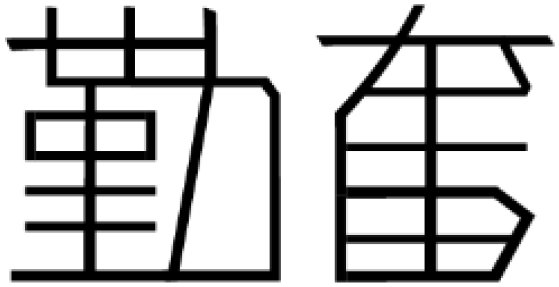	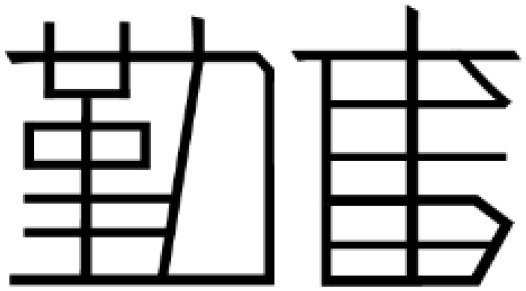

The same 48 respondents who had previously completed the association rating between high-frequency words and regular script were invited again. Under the same experimental conditions, the I_5_ reconstructed symbol schemes A, B, and C and the Chinese character “勤奮 (Qin Fen)” were presented sequentially to the respondents, who were asked to evaluate the association between the I_5_ reconstructed symbol schemes and the Chinese character “勤奮 (Qin Fen)”. The scores ranged from 1 to 5 (integer values), corresponding respectively to very difficult to recognize, difficult to recognize, average, easy to recognize, and very easy to recognize. The evaluation results, presented in Table 8, were calculated using Formula (6). The correlation values for Schemes A, B, and C are 0.257, 0.226, and 0.238, respectively. The average recognizability scores are 3.854, 3.396, and 3.563, which fall between average and easy recognition. These findings confirm that all three schemes meet the fundamental requirements for information redundancy, with Scheme A demonstrating the highest recognizability. Consequently, Scheme A is selected as the bookshelf design scheme.

**Table 8:** Correlation Evaluation of I_5_ Reconstructed Symbol Schemes and “Qin Fen” Character.

I_5_ reconstructed symbol options	Option A	Option B	Option C
**5 points**	10	3	4
**4 points**	23	17	22
**3 points**	13	24	19
**2 points**	2	4	3
**1 point**	0	0	0
$\overset{―}{p_{x}}$	0.257	0.226	0.238

### Bookshelf design integration

This bookshelf is designed as a modular, fun bookshelf for home use. Its modularity allows shelves to be placed side – by – side from left to right in Chinese character order or separated to meet diverse family needs. The bookshelf's height and size are designed to be universal, suitable for all ages, with the top shelf easily reachable. As children are the smallest height unit in a family, the bookshelf height should match children's standing reach [38]. National anthropometric data shows that the median height for 10 – year – old boys and girls is 140.1 cm and 140.2 cm, with a standing reach exceeding 160 cm. Thus, the top shelf is set at 1600 mm to meet most children's needs. The bookshelf uses two board thicknesses, reflecting the thick – thin variation in regular script calligraphy, ensuring structural solidity and a natural, vivid appearance. Given the diverse home use environments, the bookshelf serves not only for storing books but also as a decorative unit for other items. Therefore, a small section of Scheme A is designated as an enclosed storage area for less – frequently – used books and non – book items, as shown in Fig 16.

**Fig 16:**
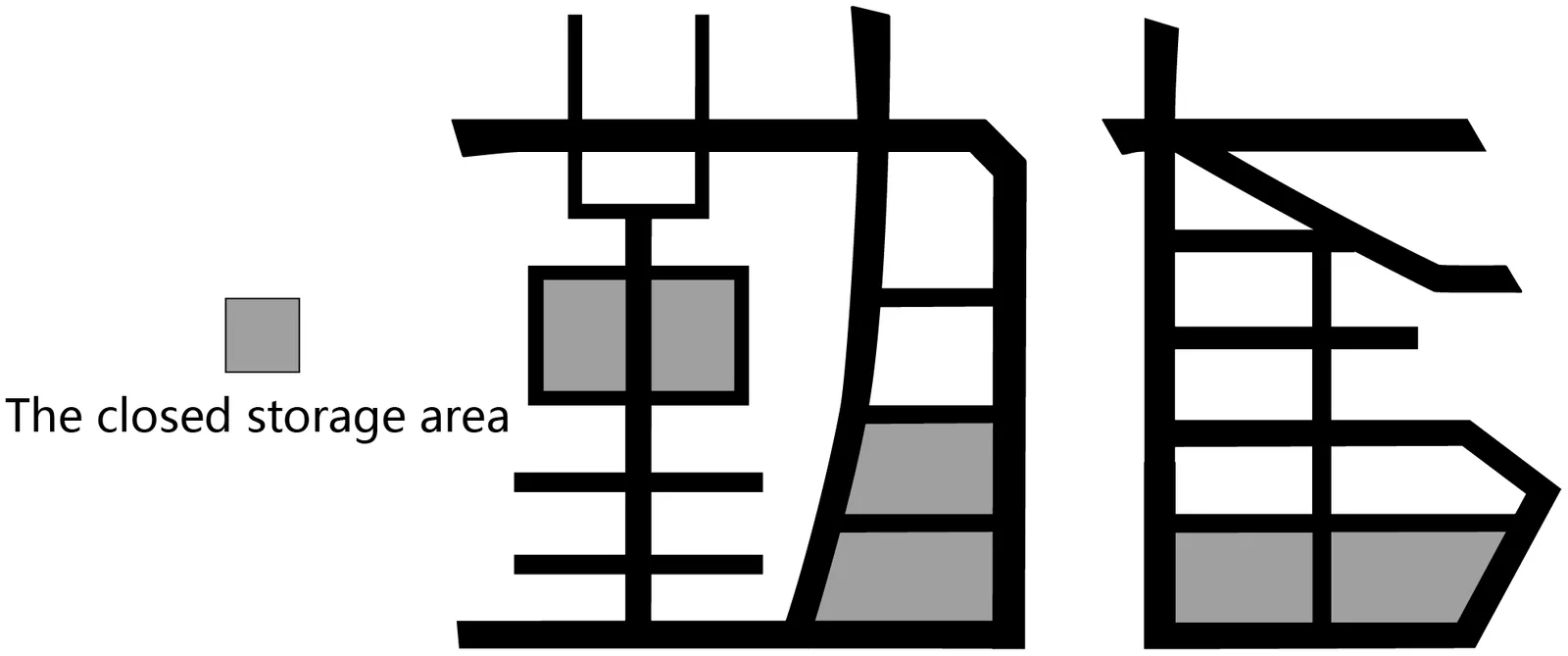
Closed Storage Area of Regular Script Bookshelf.

After multiple design iterations based on the elements in Plan A, the final two-dimensional symbol form was finalized. The 3ds Max software was used to create a three-dimensional model of the bookshelf, which was then rendered using KeyShot software. The final interior rendering is shown in Fig 17.

**Fig 17:**
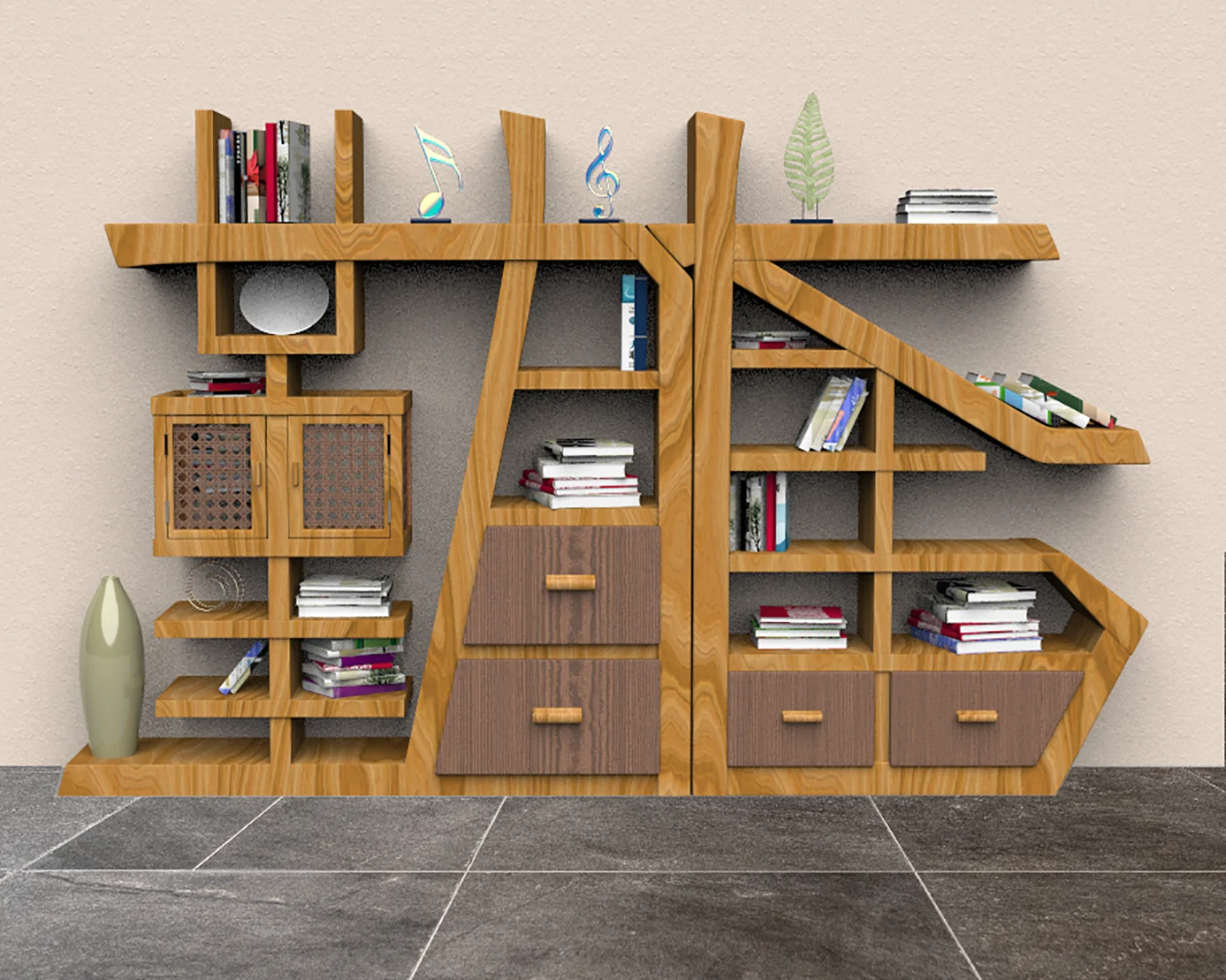
Indoor Effect Picture of Regular Script Bookshelf.

## Conclusions

As a member of the new Chinese-style furniture family, Chinese character furniture represents a continuation of traditional Chinese linear aesthetics. This study takes the “Diligence” bookshelf as a practical case to construct and validate an integrated design method for regular script furniture that combines semiotics and user needs. Through four stages—semantic extension, user-need-driven design, syntactic reconstruction, and pragmatic integration—this method systematically addresses the lack of a coherent design methodology for regular script furniture, offering a design pathway for new Chinese-style furniture that integrates cultural depth with user-oriented considerations.

The four stages of the design process correspond to the following innovative aspects:

**Semantic Extension:** A dual screening mechanism based on semantic relevance and cultural value. This ensures that the selected Chinese characters not only semantically align with the furniture but also, through quantitative evaluation of cultural design value, guarantees the characters’ design potential and innovative capacity. This overcomes the subjective and one-dimensional character selection approaches found in prior studies, strengthening the hierarchical and rational nature of the screening process.

**User-Need-Driven Design:** Systematic and hierarchical translation of user needs into design parameters. Key user needs were collected and refined using the KJ method, and their priorities were ranked through vector relationship analysis. These needs were then mapped into specific, uncoupled design parameters via Axiomatic Design theory. This stage translates users’ vague, perceptual intentions into concrete operational instructions through logical reasoning.

**Syntactic Reconstruction:** A morphology derivation method guided by design parameters. Under the direction of these parameters, regular script strokes were encoded, and shape grammar rules were iteratively applied to evolve the script symbols until a satisfactory form was achieved, simultaneously generating diverse design alternatives. This approach of translating design requirements into shape generation enhances the acceptability of the design proposals, as it balances appropriate stroke variation with basic recognizability, preserving the aesthetic essence of regular script while accommodating constraints of manufacturing and economic feasibility.

**Pragmatic Integration:** In-depth translation of reconstructed regular script symbols into specific product parameters. Guided by usage scenarios and based on user anthropometric data, material properties, and functional requirements—combined with the spatial enclosure and stroke flow of the reconstructed script symbols—an iterative process yielded a new furniture product that harmonizes tradition and novelty, practicality and aesthetics, legibility and cultural depth.

From the perspective of the bookshelf design case, it has become a representation of the productization of regular script symbols. The bookshelf’s form not only aligns with the MAYA (Most Advanced Yet Acceptable) principle, but its moderate variation also stimulates curiosity and interest in the audience. Local details of the bookshelf capture the brushstroke effect of regular script, concise yet rich in calligraphic charm, highlighting Chinese aesthetics while resonating with contemporary tastes. As users interact with or utilize the bookshelf, they decode the semantic message of the Chinese characters “Diligence,” thereby subtly reinforcing the educational function of the furniture.

This study provides an example and suggests directions for the integrated application of research methods within the framework of design semiotics. The proposed process offers clear operational guidelines for the design of regular script and Chinese character furniture, expands the research domain of regular script within new Chinese-style furniture, and provides a systematic, innovative, and user-oriented reference for research and design related to the modern translation of traditional symbols.

**Limitations of this study:** Although the bookshelf case validates the feasibility of the design process, it remains a single case study. More design cases are needed to further verify the generalizability of the process and to identify potential, currently unknown issues.

The design process developed in this study is primarily constructed for regular script. Future research could explore the systematic expression of furniture based on other calligraphic styles, in response to the evolving aesthetic and emotional needs of people. For instance, the flowing and connected strokes of running script and cursive script convey a sense of elegance and fluidity, which could be translated into the artistic language of furniture. Compared to the regular, square structure of regular script, seal script and clerical script exhibit a softer, rounded-square quality, giving a “softer” visual impression that could be transformed into a design language expressing humanistic care in furniture.

## Supporting information

S1 Fig Reachability matrix calculation. (TIF)

S1 File KJ method analysis of user needs. (PPTX)

S2 File Correlation Evaluation between High-Frequency Words and Regular Script-raw date. (XLSX)

S3 File Cultural Design Value Assessment of High-Frequency Words (Simplified and Traditional)-raw date. (XLSX)

S4 File Bookshelf Semantic Association Survey Questionnaire -raw data. (XLSX)

S5 File Correlation Evaluation of Reconstructed Symbol Schemes and Qin Fen Character. (XLSX)

S6 File PPT on the Assessment of Extensible Semantic Analysis. (PDF)
