# Supplementary material for: Integrated design process of Chinese character furniture through regular script
Source: PLoS One. 2026 Oct 26;21(10):e0360938. doi: 10.1371/journal.pone.0360938 (PMC13704814; doi:10.1371/journal.pone.0360938)

可拓语义关联性评价说明(Explanation of the Correlation Evaluation in Extensible Semantic Analysis)

向受访者说明关联性评价：量化候选汉字与楷书本体之间形式及文化关联强度的指标。
Explanation of Correlation Evaluation to Respondents: This evaluation is defined as a quantitative metric that measures the strength of both formal (visual/morphological) and cultural correlation between candidate Chinese characters and Regular Script (Kǎishū) in this study.

邀请48位受访者对高频字词与楷书进行关联性评价。

A total of 48 respondents were invited to conduct a correlation evaluation between the high-frequency characters and Regular Script (Kǎi-shū).

打分1-5分，取整数，取值越高表示二者的关联性越高，反之，则越低。

Scores are given on a scale of 1 to 5 points, using integers, where a higher score indicates a stronger correlation between the two, and a lower score indicates a weaker correlation.

请将“书(Shu)” 与楷书进行关联性打分。

Please rate the character "书(Shu)" on its correlation with Regular Script (Kǎishū).

打分取值1-5分

Scoring ranges from 1 to 5 points.

"书(Shu)"
Character

（ ）

“楷书(Kaishu)“
Regular Script

请将“知识(Zhi Shi)" 与楷书进行关联性打分。

Please rate the character "知识(Zhi Shi)" on its correlation with Regular Script (Kǎishū).

打分取值1-5分

Scoring ranges from 1 to 5 points.

"知识(Zhi Shi)" Character ←（　　）→ “楷书(Kai Shu)“ Regular Script

请将“学习(Xue Xi)" 与楷书进行关联性打分。

Please rate the character "学习(Xue Xi)" on its correlation with Regular Script (Kǎishū).

打分取值1-5分

Scoring ranges from 1 to 5 points.

"学习(Xue Xi)" Character ←（　　）→ “楷书(Kai Shu)“ Regular Script

请将“勤奋(Qin Fen)" 与楷书进行关联性打分。

Please rate the character "勤奋(Qin Fen)" on its correlation with Regular Script (Kǎishū).

打分取值1-5分

Scoring ranges from 1 to 5 points.

"勤奋(Qin Fen)" Character ( ) ←→ “楷书(Kai Shu)“ Regular Script

请将“努力(Nu Li)"与楷书进行关联性打分。

Please rate the character "努力(Nu Li)"on its correlation with Regular Script (Kǎishū).

打分取值1-5分

Scoring ranges from 1 to 5 points.

"努力(Nu Li)"
Character

（　　）

“楷书(Kai Shu)“
Regular Script

可拓语义文化设计价值评价说明(Explanation of Cultural Design Value Assessment in Extensible Semantic Analysis)

向受访者说明文化设计价值评估：它特指一个汉字作为设计原型时，其**文化内涵的强度、美学转化潜力及与产品功能的叙事耦合度**的综合潜力。

Explanation of Cultural Design Value Assessment to Respondents: This assessment refers specifically to the comprehensive potential of a Chinese character when used as a design prototype, encompassing the strength of its cultural connotations, its potential for aesthetic transformation, and its narrative coupling with product functions.

邀请25位具有家具设计经验且对楷书颇具了解的设计师和教师，作为受访者对上述5个高频字词进行文化设计价值评估。Twenty-five designers and instructors with furniture design experience and a solid understanding of Regular Script (Kǎishū) were invited as respondents to assess the cultural design value of the aforementioned five high-frequency characters.

分值取值为1~7分，取整数，评分越高表示文化设计价值越大，反之，则越小。

Scores were given on a scale of 1 to 7 points, using integers, where a higher score indicates a greater cultural design value, and a lower score indicates a smaller one.

请评价"书(Shu)" 的文化设计价值。

Please evaluate the cultural design value of the "书 (Shu)" (Simplified Character).

打分取值1-7分

Scoring ranges from 1 to 7 points.

"书(Shu)" Simplified Character

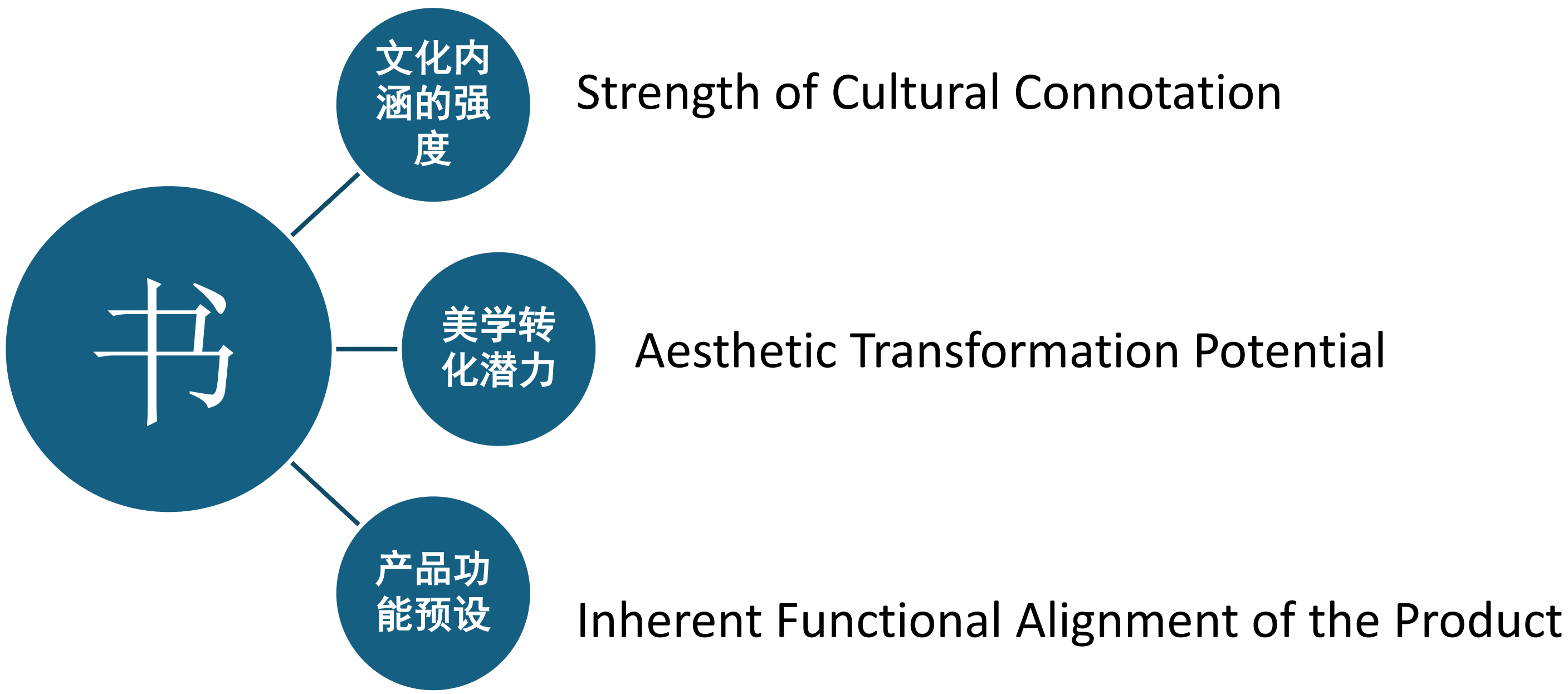

请评价"書(Shu)" 的文化设计价值。

Please evaluate the cultural design value of the "书 (Shu)" (Simplified Character).

打分取值1-7分

Scoring ranges from 1 to 7 points.

"書(Shu)" Simplified Character

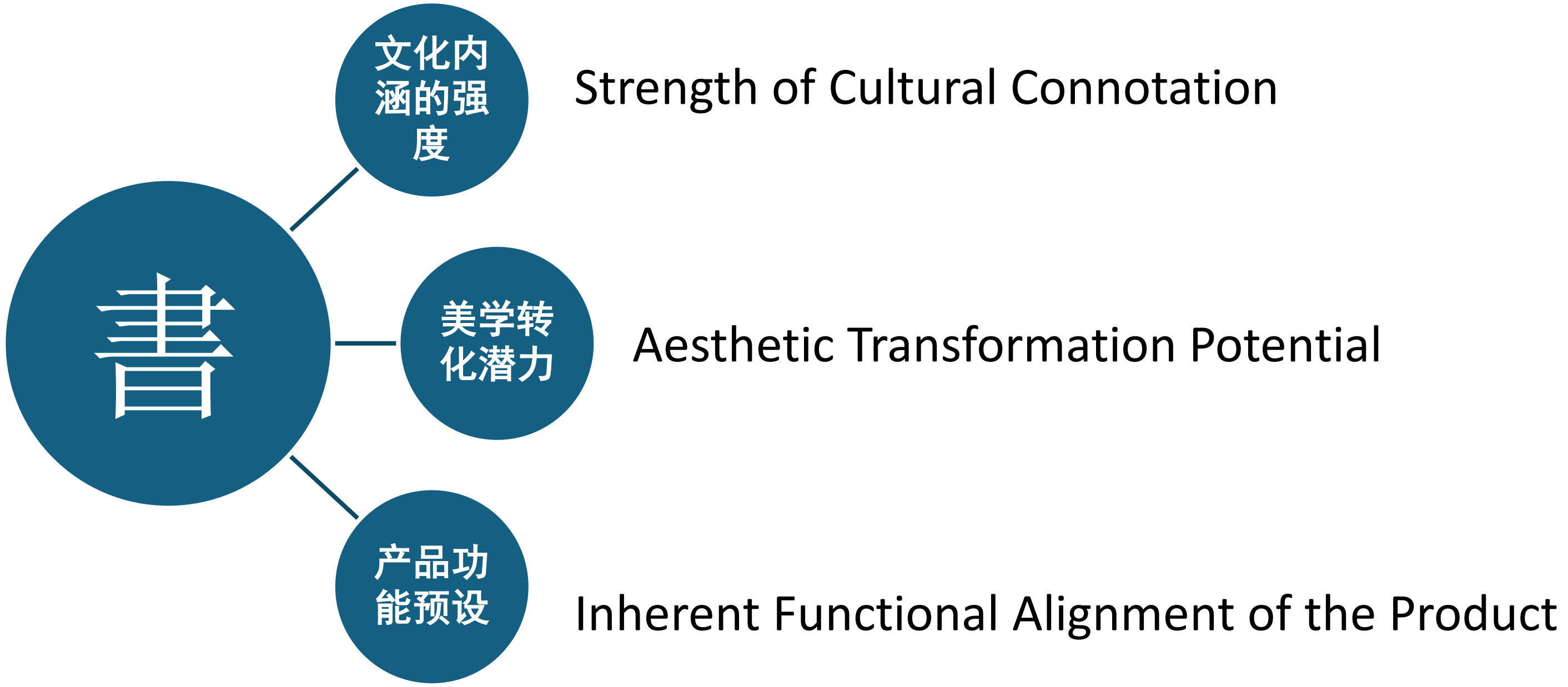

请评价"知识(Zhi Shi) "的文化设计价值。

Please evaluate the cultural design value of the "书 (Shu)" (Simplified Character).

打分取值1-7分

Scoring ranges from 1 to 7 points.

"知识(Zhi Shi)" Simplified Character

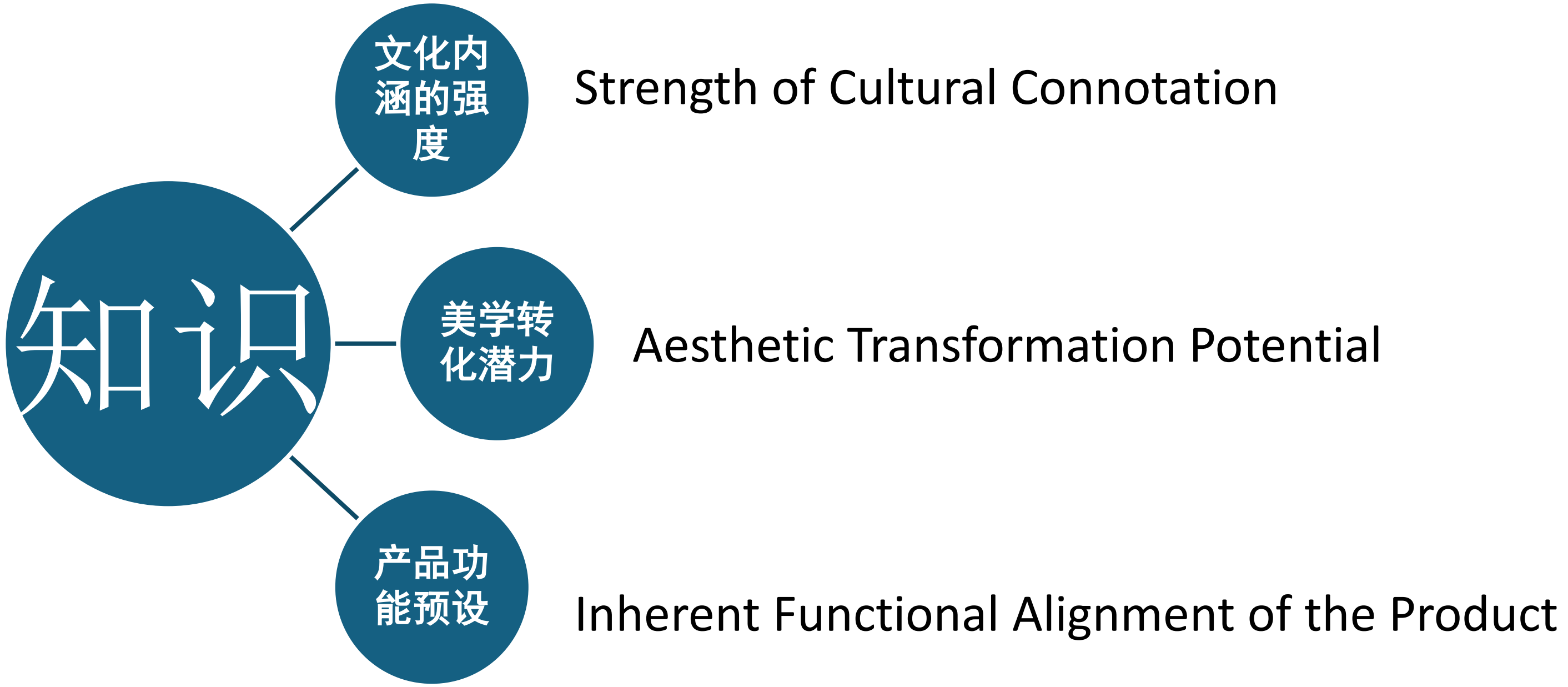

请评价"知識(Zhi Shi) "的文化设计价值。

Please evaluate the cultural design value of the "书 (Shu)" (Simplified Character).

打分取值1-7分

Scoring ranges from 1 to 7 points.

"知識(Zhi Shi)" Simplified Character

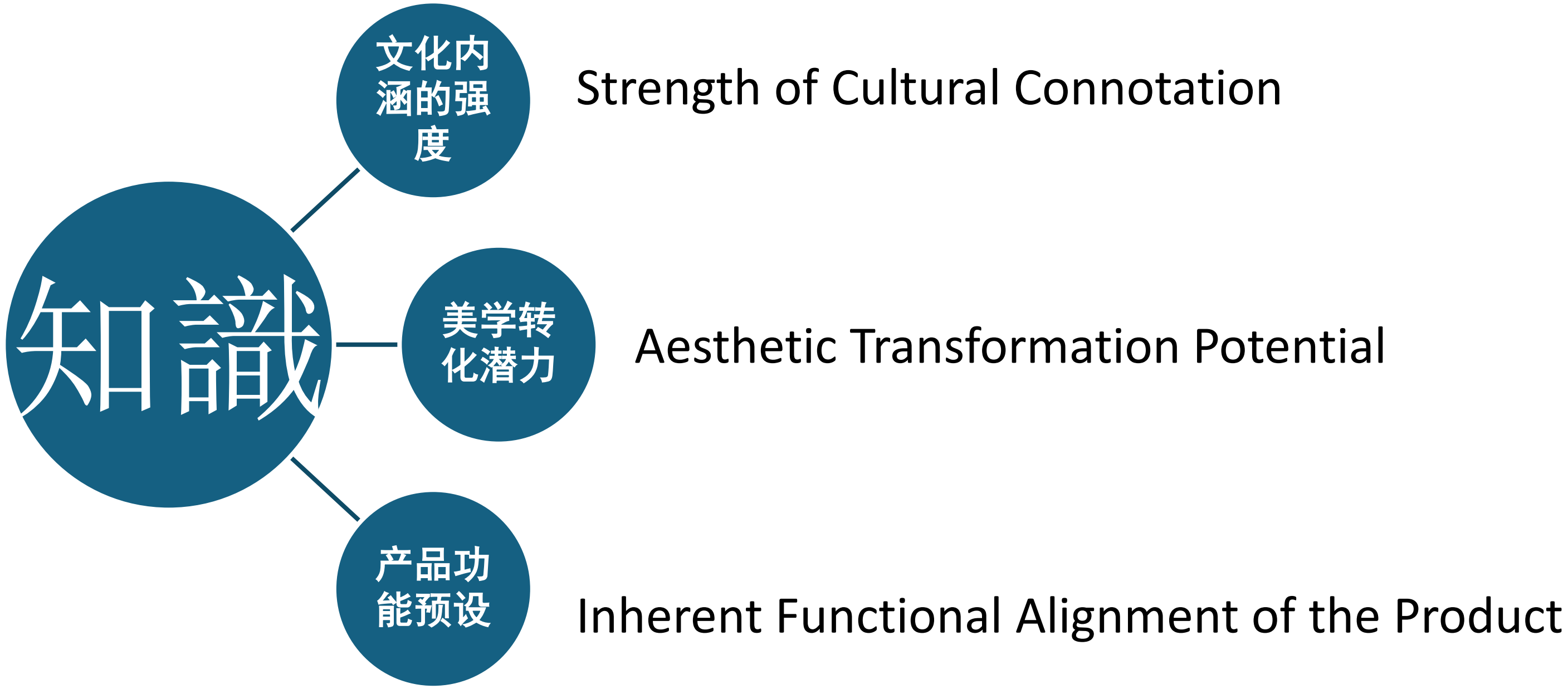

请评价"学习(Xue Xi)"的文化设计价值。

Please evaluate the cultural design value of the "书 (Shu)" (Simplified Character).

打分取值1-7分

Scoring ranges from 1 to 7 points.

"学习(Xue Xi)" Simplified Character

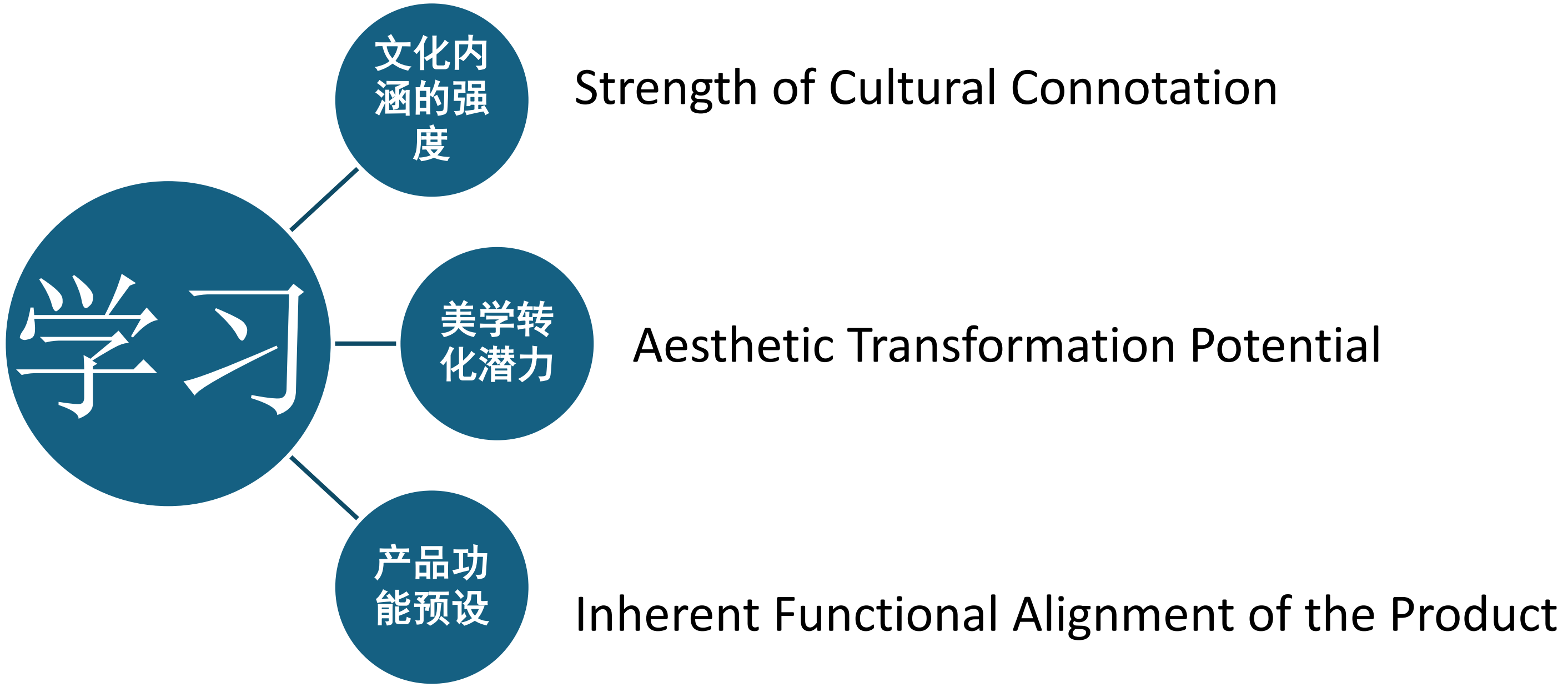

请评价"學習(Xue Xi)"的文化设计价值。

Please evaluate the cultural design value of the "书 (Shu)" (Simplified Character).

打分取值1-7分

Scoring ranges from 1 to 7 points.

"學習(Xue Xi)" Simplified Character

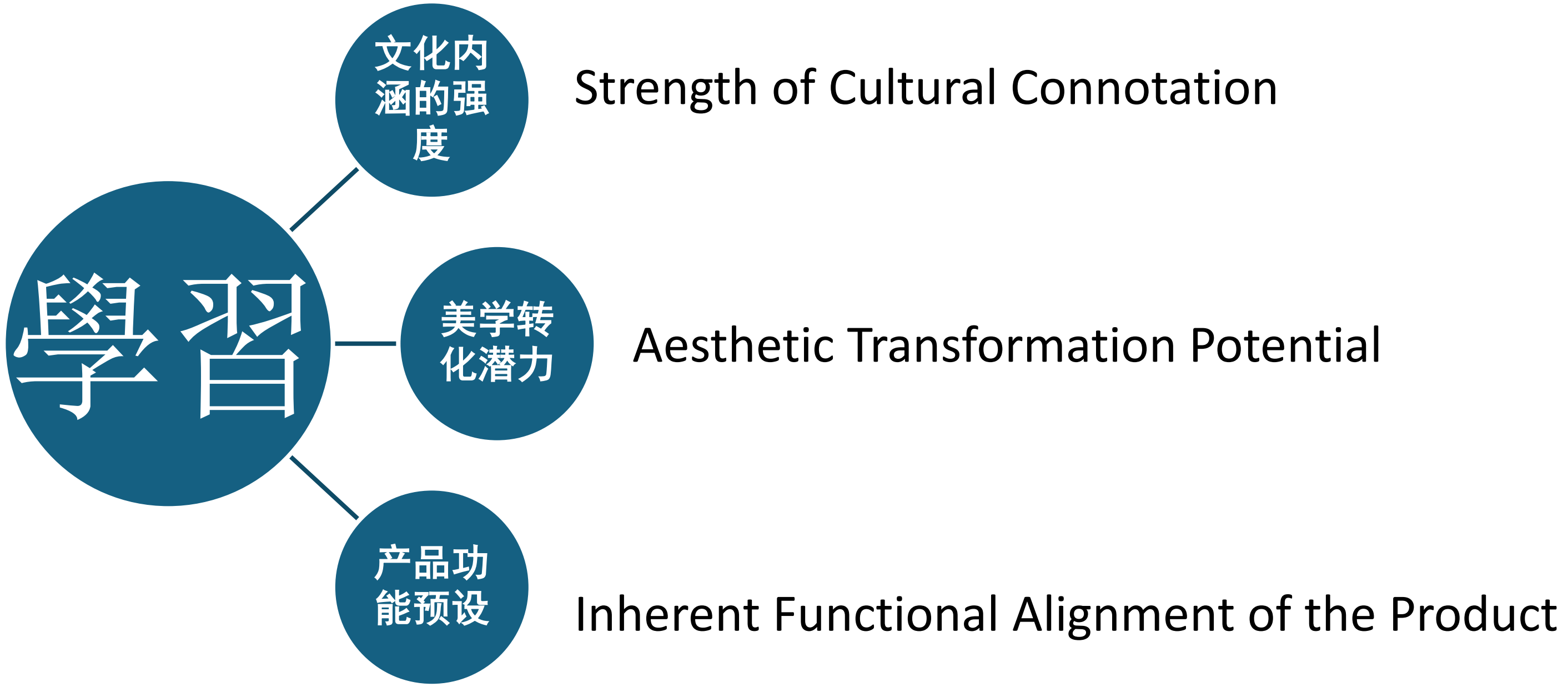

请评价"勤奋(Qin Fen)"的文化设计价值。

Please evaluate the cultural design value of the "书 (Shu)" (Simplified Character).

打分取值1-7分

Scoring ranges from 1 to 7 points.

"勤奋(Qin Fen)" Simplified Character

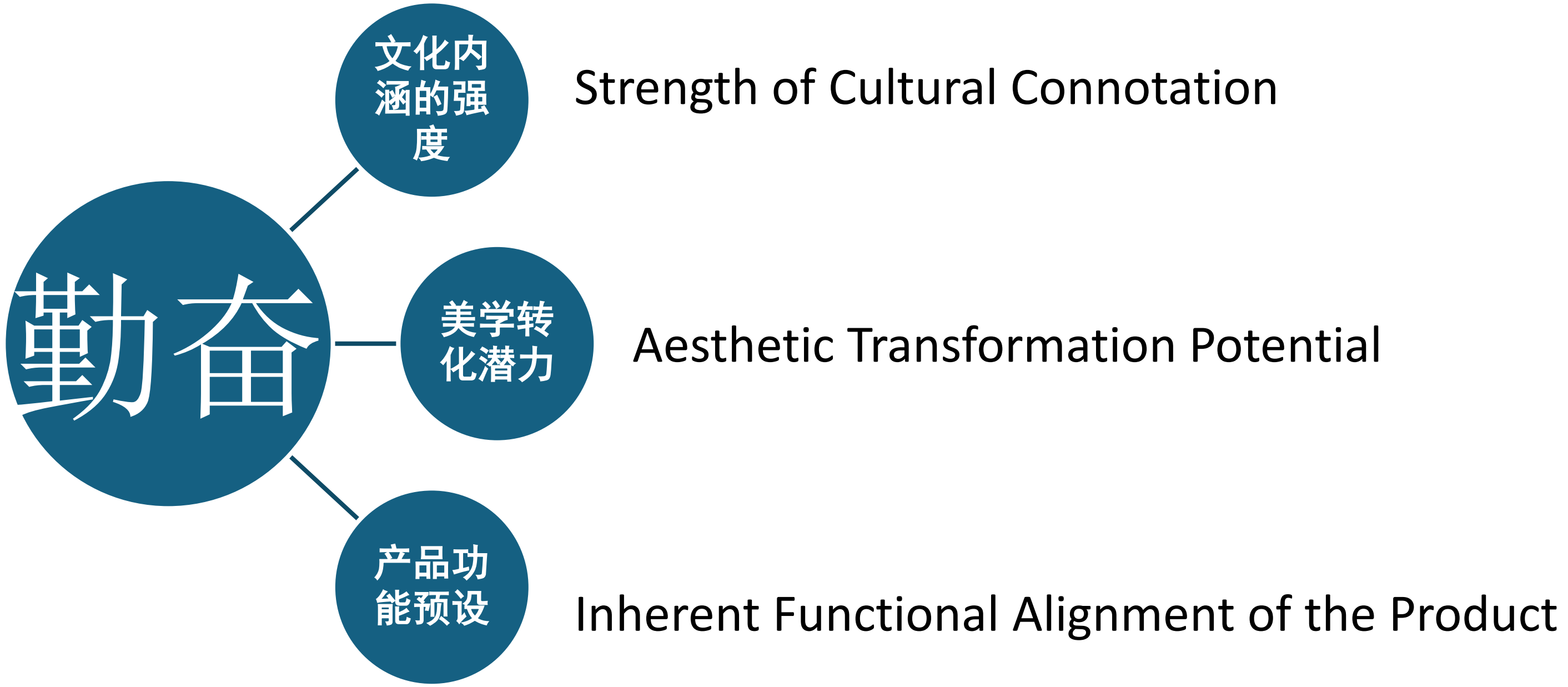

请评价"勤奮(Qin Fen)"的文化设计价值。

Please evaluate the cultural design value of the "书 (Shu)" (Simplified Character).

打分取值1-7分

Scoring ranges from 1 to 7 points.

"勤奮(Qin Fen)" Simplified Character

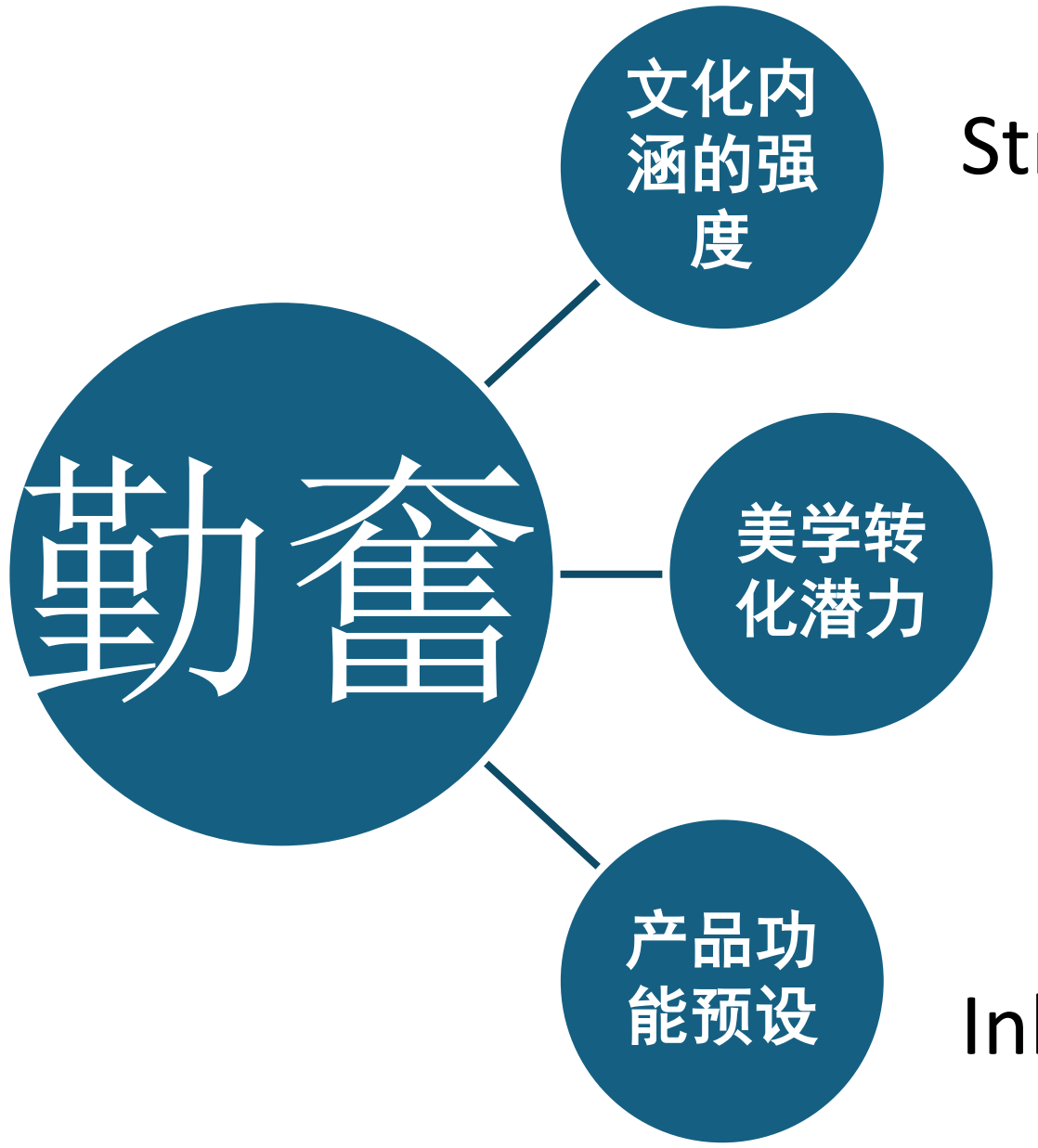

Strength of Cultural Connotation

Aesthetic Transformation Potential

Inherent Functional Alignment of the Product

请评价"努力(Nu Li)"的文化设计价值。

Please evaluate the cultural design value of the "书 (Shu)" (Simplified Character).

打分取值1-7分

Scoring ranges from 1 to 7 points.

"努力(Nu Li)"Simplified Character

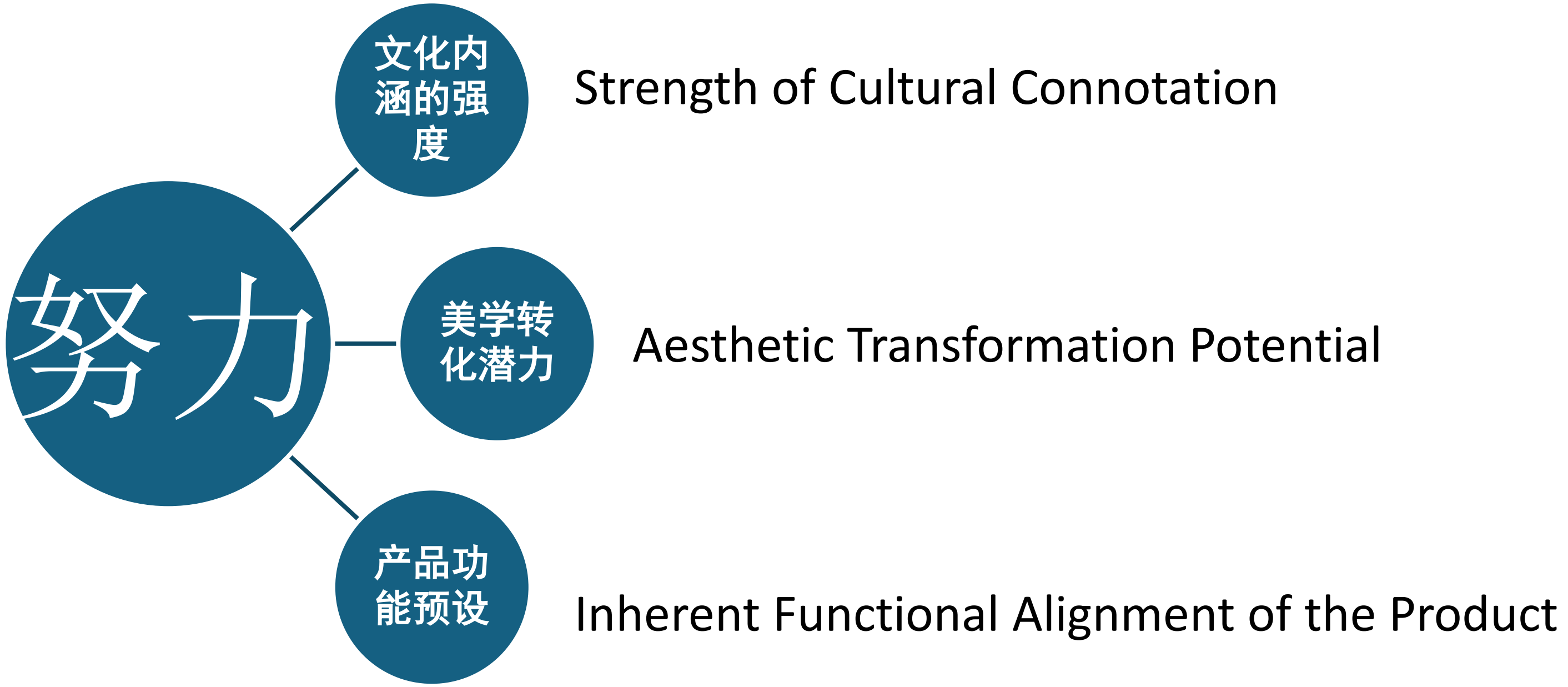

重构符号方案与“勤奮”汉字可拓语义关联性评价 (Correlation Evaluation in Extensible Semantic Analysis between the Reconstructed Symbol Options and the "Diligence (勤奮)" Chinese Character)

邀请上述48位已经完成高频字词与楷书关联性评价的受访者，对I_5重构符号方案与“勤奮”汉字进行关联性评价。

The aforementioned 48 respondents, who had already completed the correlation evaluation between high-frequency words and Regular Script, were invited to perform a correlation evaluation between the I_5 reconstructed symbol options and the "Diligence (勤奮)" Chinese character.

分值为1-5分，分别对应非常不容易辨认、不容易辨认、一般、容易辨认、非常容易辨认。

Scores range from 1 to 5 points, corresponding respectively to: very difficult to recognize, difficult to recognize, average, easy to recognize, and very easy to recognize.

请将“勤奮”汉字符号与楷书重构方案A进行关联性打分。

Please rate the correlation between the Chinese character symbol "Diligence (勤奮)" and Regular Script Reconstructed Scheme A.

打分取值1-5分

Scoring ranges from 1 to 5 points.

The Regular script symbol of "Diligence"

勤奮

（　　）

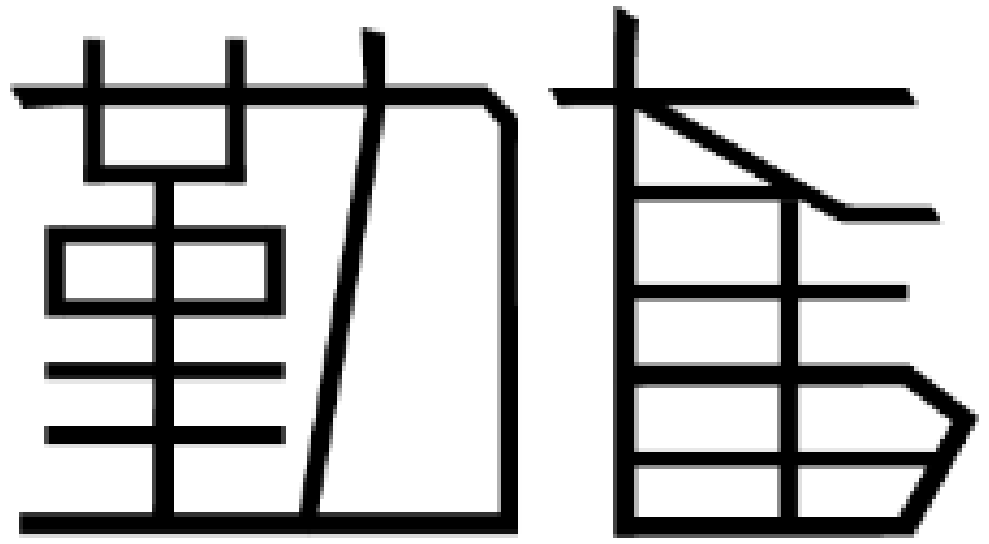

请将“勤奮”汉字符号与楷书重构方案B进行关联性打分。

Please rate the correlation between the Chinese character symbol "Diligence (勤奮)" and Regular Script Reconstructed Scheme B.

打分取值1-5分

Scoring ranges from 1 to 5 points.

The Regular script symbol of "Diligence"

勤奮

（   ）

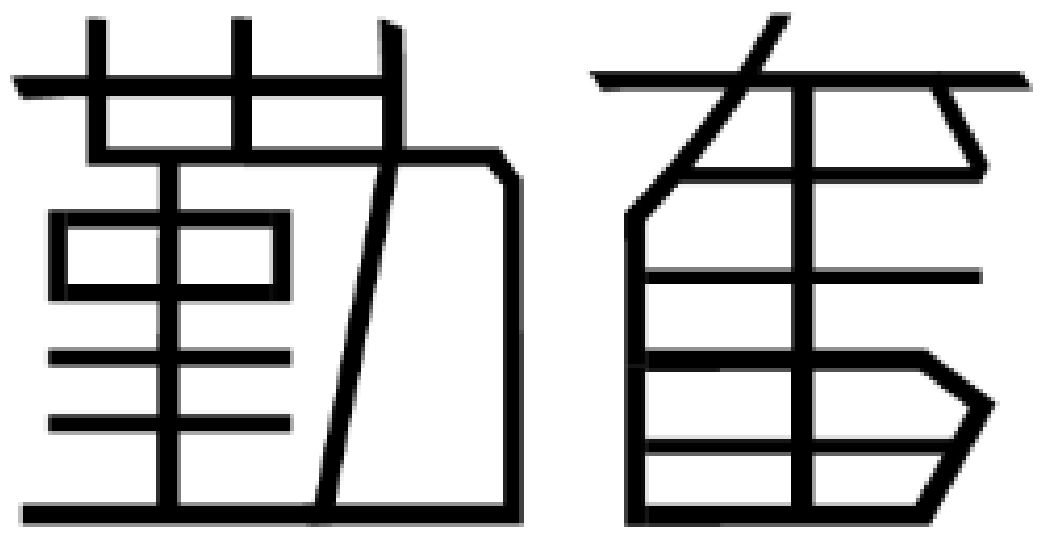

请将“勤奮”汉字符号与楷书重构方案C进行关联性打分。

Please rate the correlation between the Chinese character symbol "Diligence (勤奮)" and Regular Script Reconstructed Scheme C.

打分取值1-5分

Scoring ranges from 1 to 5 points.

The Regular script symbol of "Diligence"

勤奮

（　　）

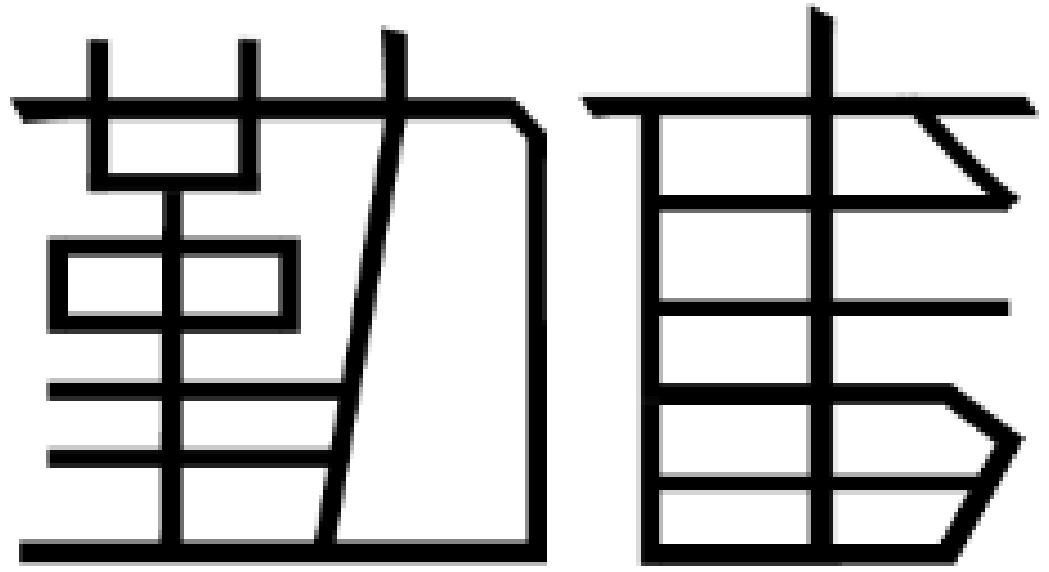

Supplement: Supplementary file 1 [file pone.0360938.s007.pdf]
